# Wnt, Notch, and TGF-β Pathways Impinge on Hedgehog Signaling Complexity: An Open Window on Cancer

**DOI:** 10.3389/fgene.2019.00711

**Published:** 2019-08-21

**Authors:** Maria Pelullo, Sabrina Zema, Francesca Nardozza, Saula Checquolo, Isabella Screpanti, Diana Bellavia

**Affiliations:** ^1^Center of Life Nano Science Sapienza, Istituto Italiano di Tecnologia, Rome, Italy; ^2^Department of Molecular Medicine, Sapienza University, Rome, Italy; ^3^Department of Medico-Surgical Sciences and Biotechnologies, Sapienza University, Latina, Italy

**Keywords:** Hedgehog, Notch, Wnt, TGF-β, signaling pathway, tumorigenesis

## Abstract

Constitutive activation of the Hedgehog (Hh) signaling pathway is associated with increased risk of developing several malignancies. The biological and pathogenic importance of Hh signaling emphasizes the need to control its action tightly, both physiologically and therapeutically. Evidence of crosstalk between Hh and other signaling pathways is reported in many tumor types. Here, we provide an overview of the current knowledge about the communication between Hh and major signaling pathways, such as Notch, Wnt, and transforming growth factor β (TGF-β), which play critical roles in both embryonic and adult life. When these pathways are unbalanced, impaired crosstalk contributes to disease development. It is reported that more than one of these pathways are active in different type of tumors, at the same time. Therefore, starting from a plethora of stimuli that activate multiple signaling pathways, we describe the signals that preferentially converge on the Hh signaling cascade that influence its activity. Moreover, we highlight several connection points between Hh and Notch, Wnt, or TGF-β pathways, showing a reciprocal synergism that contributes to tumorigenesis, supporting a more malignant behavior by tumor cells, such as in leukemia and brain tumors. Understanding the importance of these molecular interlinking networks will provide a rational basis for combined anticancer drug development.

## Introduction

Signaling pathways are networks of regulatory proteins and other gene products that act in a coordinated manner to control various biological processes inside the cell. Remarkably, mutation of a single gene encoding a component of a specific pathway is able to affect related signaling cascades, triggering unbalanced crosstalk that leads to cancer development. Of note, impaired regulation of primary signaling pathways can ultimately culminate in constitutive activation of signaling effectors in the nucleus, where they act out of control, sustaining the expression of pro-tumoral target genes. To date, it is known that tumor development is characterized by deregulation of at least two major signaling pathways at the same time, which crosstalk with each other, determining the acquisition of malignant phenotypes (Petrova and Joyner, 2014).

Hedgehog (Hh) signaling is a critical pathway that mainly controls embryonic development, whereas in post-natal life, it is inactive or poorly active, playing a restricted role in stem cell maintenance and tissue homeostasis/repair (Petrova and Joyner, 2014). Since Hh signaling regulates a wide array of biological activities in various cell types, its misregulation is responsible for the development of many types of cancers. Studies show that the mutational activation of Hh signaling is a nodal point in sustaining tumorigenic programs, ranging from the tumor initiation to tissue invasion/metastasis and chemoresistance, in several different tumors.

Of note, recent studies show that Hh signaling elements talk to several other cofactors belonging to major pathways, such as Notch, Wnt, and transforming growth factor β (TGF-β), resulting in significant crosstalk between these signaling networks. The integration of several signaling pathways is a key step able to determine a more aggressive behavior of tumor cells and their resistance to pharmacological approaches. Interestingly, Notch, Wnt, and TGF-β pathways are able to promote/sustain oncogenesis through synergistic associations with Hh signaling in several types of tumors.

Here, we review a global picture of intricate protein–protein interaction networks between key components of Hedgehog, Wnt, Notch, and TGF-β signaling pathways in an unbalanced/malignant context. We also describe how these main pathways can integrate with each other and ultimately affect Hh signaling output.

## The Hedgehog Pathway

**Figure 1:**
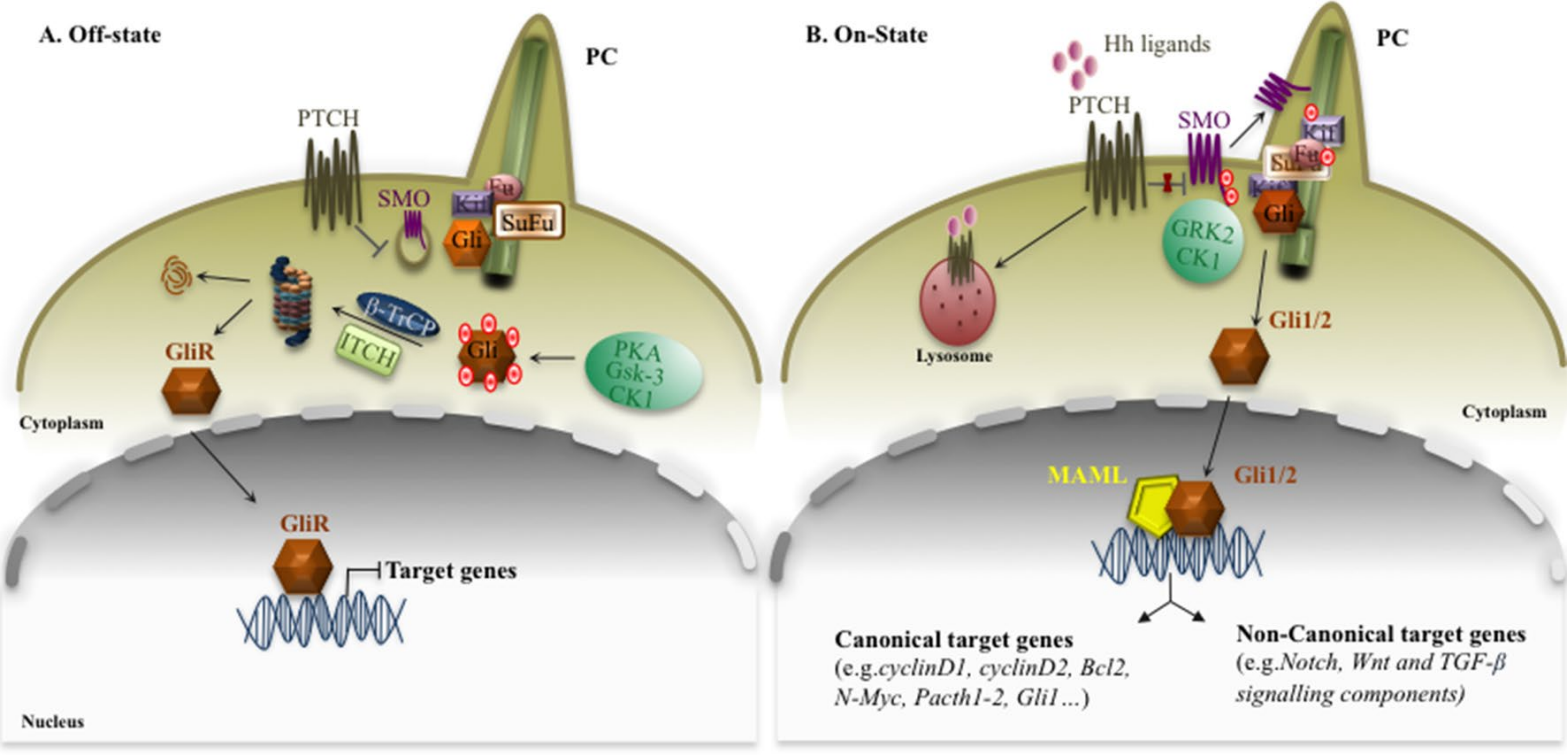
Canonical Hh signaling. The canonical Hh signaling pathway in off (panel **A**) or on (panel **B**) state. The figure is widely discussed in the text. The red dots represent post-translational modifications: phosphorylation.

First discovered in *Drosophila*, Hedgehog signaling is an evolutionarily conserved pathway that plays a key role as a morphogenesis driver for embryonic and post-natal development. It regulates diverse cellular processes, including cell proliferation, tissue differentiation, and repair of normal tissues (Nusslein-Volhard and Wieschaus, 1980; Napolitano et al., 1999; Varjosalo and Taipale, 2008), and it is also implicated in regulation/survival of normal and malignant stem cells (Liu et al., 2006; Merchant and Matsui, 2010). Canonical Hedgehog pathway activation is characterized by the interaction of Hh ligands, Sonic (SHh), Indian (IHh), and Desert (DHh), to the Patched1 (Ptch1) receptor, which resides in the primary cilium (PC) (Rohatgi et al., 2007; Wong and Reiter, 2008). Interestingly, the PC is a key organelle that consists of microtubules emanating from the cell surface in which SHh signaling takes place, and it responds to mechanical stimuli in the micro-environment (Michaud and Yoder, 2006). In the absence of Hh ligand, Ptch localizes to the base of the PC and catalytically represses the activity of the transducer Smoothened (SMO) (Burns et al., 2018), a member of G-protein–coupled receptor-like (GPCR), by inhibiting its translocation into the PC (Denef et al., 2000) (**Figure 1A**). SMO is a seven-transmembrane-span receptor-like protein that is confined to intracellular endocytic vesicles when the Hh pathway is switched off (Taipale et al., 2002). Hh binding both causes the internalization of ligand/receptor complex from the cell surface towards lysosomes, where the proteins are degraded (Mastronardi et al., 2000), and promotes the accumulation of SMO at the cell surface (Denef et al., 2000). Once activated, SMO is hyperphosphorylated by casein kinase 1 (CK1) and G-protein–coupled receptor kinase 2 (GRK2), resulting in the release of its inhibition, and at this point, it is free to move from the base into the tip of the PC (Denef et al., 2000; Chen et al., 2011) (**Figure 1B**). Given these features, SMO is considered a positive regulator of the Hh signaling pathway because, when it is constitutively activated, it stimulates downstream components of the signaling pathway. Therefore, the ligand/receptor complex relieves the SMO inhibition and triggers a cascade of intracellular processes that involve a dynamic association between Gli transcription factors, the final effectors of Hh signaling, and Suppressor of Fused (SuFu). Unlike SMO, SuFu is considered a tumor suppressor gene and a negative regulator of Hedgehog signaling, able to bind directly to Gli proteins to regulate their activity, processing, and localization, (Ryan and Chiang, 2012), by sequestering them in the cytosol (Ding et al., 1999; Dunaeva et al., 2003) or regulating their affinity to DNA (Pearse et al., 1999; Stone et al., 1999; Sisson et al., 2006). However, the specific mechanisms concerning Gli inactivation by SuFu are not completely understood (Carballo et al., 2018). Hh ligand binding sustains the release of Gli from SuFu that moves into the nucleus and activates Hh target genes. Altogether, these molecular events sustain the signal that is transduced from the ligand/receptor complex to the downstream transcription factors (Gli1, Gli2, and Gli3), which in turn translocate into the nucleus and regulate the expression of Hh target genes, including Gli1 itself. Prominently, Gli1 is both the downstream effector and a target gene of the pathway, representing a feedback loop that serves as a readout of Hh activity (Sasaki et al., 1999; Regl et al., 2002). Interestingly, Gli1 consists of zinc finger and C-terminal activator domains, whereas Gli2 and Gli3 also possess an N-terminal repressor domain. On the basis of these structural differences, Gli1 functions exclusively as a transcriptional activator, whereas Gli2 and Gli3 exist as full-length (FL) activator and truncated repressor (GliR) forms, displaying in this way both positive and negative transcriptional functions (Lee et al., 1997; Dai et al., 1999; Sasaki et al., 1999; Bai et al., 2004). In the absence of Hh ligand, GliFL is phosphorylated by protein kinase-A (PKA), glycogen synthase kinase-3 (GSK3), and CK1 (Price and Kalderon, 2002), and recognized by β-transducin-repeat containing protein (β-TrCP), which is able to partially degrade Gli proteins in truncated forms (Glis). At this point, Gli proteins retained at the cytoplasm by SuFu are degraded, inhibiting SHh signaling (Humke et al., 2010). These events lead to proteolytic cleavage of GliFL into C-terminally truncated repressor form, GliR, which translocates to the nucleus, where it binds to Hh target gene promoters and keeps them switched off (Goetz and Anderson, 2010). Interestingly, a recent paper shows that the Itch/β-arrestin2 complex binds SuFu and induces its polyubiquitination without any impact on stability, but sustaining the conversion of Gli3 into a repressor, which is able to switch off the signal (Infante et al., 2018).

Under basal conditions, Gli1 activity can be controlled by proteasome degradation, mediated by two distinct ubiquitin-dependent processing pathway E3 ubiquitin ligases, β-TrCP and Itch, able to inactive Gli1 in the cytosol (Lee et al., 1997; Di Marcotullio et al., 2006; Di Marcotullio et al., 2007; Di Marcotullio et al., 2011; Infante et al., 2016). On the contrary, the activation of Hh/Gli signaling is associated with a translocation of Gli1 into the nucleus, where it exerts strong mitogenic and prosurvival activities. Another mechanism able to control the Hh signaling is based on the acetylation status of Gli1 and Gli2, mediated by Histone deacetylases (HDACs). Indeed, HDAC-mediated deacetylation promotes transcriptional activation and sustains a positive regulatory loop. This mechanism is turned off by HDAC1 degradation, mediated by a Cullin3/Ren complex (Canettieri et al., 2010).

Recent studies identify Mastermind-like 1 (Maml1) as a novel positive regulator of Hh signaling. It physically interacts with Gli proteins (Gli1 and Gli2), working as a potent transcriptional coactivator (Quaranta et al., 2017; Vied and Kalderon, 2009), empowering Gli-mediated transcriptional activity. Finally, Gli1 activation induces the transcription of Hh target genes involved in key cellular processes, such as the cell cycle (Cyclin D1 and D2) (Sasaki et al., 1999), apoptosis (Bcl2) (Bigelow et al., 2004), N-Myc (Oliver et al., 2003), various transcription factors and components of the Hh pathway itself such as *Ptch1*, *Ptch2*, and Gli1 for negative and positive feedback loop mechanisms (canonical target genes) (Bonifas et al., 2001; Oliver et al., 2003, Vokes et al., 2007; Coni et al., 2013), and elements of other pathways such as Notch1 and Jagged1 (Stecca and Ruiz i Altaba, 2009), Hes1 (Ingram et al., 2008; Wall et al., 2009), Wnt signals (Mullor et al., 2001), and TGF-β members (Fan et al., 2010) (non-canonical target genes), suggesting that the Hh signaling pathway can integrate with elements of major signaling pathways (**Figure 1**).

## Hedgehog Mutations and Disease

Mutational activation of the Hh signaling pathway is tightly linked to maintenance of tumor-initiating stem cells, tumorigenesis, and tumor invasiveness in several types of cancer (Nilsson et al., 2000; Varnat et al., 2009; Harris et al., 2012). Mutations in Hh signaling can be classified as loss of function (i.e., *Ptch1* and *SuFu*) or gain of function (i.e., *Gli1*, *Gli2*, and *SMO*), both associated with an aberrant activation of the Hh pathway (**Table 1**). Constitutive Hh signaling triggers a strong cellular mitogenic response that ultimately predisposes to abnormal proliferation leading to tumorigenesis.

**Table 1:** Mutations of HH signaling

	Gene	Locus	Disease	Inheritance	Mutations	Reference
Loss of function	Ptch1	9q22.32	Gorlin syndrome	Germline	Insertion deletion	Hahn et al., 1996; Lindstrom et al., 2006
			Rhabdomyosarcoma	Germline	Missense	Bridge et al., 2000; Taeubner et al., 2018
			Medulloblastoma	Germline Somatic	Insertion Deletion Frameshift LOH	Raffel et al., 1997; Reifenberger et al., 1998; Zurawel et al., 2000; Thompson et al., 2006; Kool et al., 2014; Rusert et al., 2014
			Basal cell carcinoma	Somatic	Missense LOH	Gailani et al., 1996; Reifenberger et al., 2005
			Leukemia	Germline Somatic	Missense	Dagklis et al., 2015; Burns et al., 2018
			Breast cancer	NA	LOH	Naylor et al., 2005; Wood et al., 2007; Sinha et al., 2008
			Gastric–intestinal cancer	NA	Frameshift Missense	Wang et al., 2013
			Gorlin syndrome	Germline	Missense Frameshift	Fan and Eberhart, 2008; Fujii et al., 2013
	Ptch2	1p34.1	Rhabdomyosarcoma	Germline	Missense	Taeubner et al., 2018
			Leukemia	NA	Transition	Dagklis et al., 2015
	SuFu	10q24.32	Gorlin syndrome	Germline	Nonsense Missense deletion	Smith et al., 2014
			Rhabdomyosarcoma	Germline	LOH	Bridge et al., 2000
			Medulloblastoma	Germline Somatic	Missense deletion Truncating LOH	Taylor et al., 2002; Thompson et al., 2006; Rusert et al., 2014; Kool et al., 2014
			Leukemia	Somatic	Missense	Burns et al., 2018
			Basal cell carcinoma	Somatic	Missense	Reifenberger et al., 2005
			Prostate cancer	Somatic	LOH deletion Nonsense	Sheng et al., 2004
			Meningioma	Germline	Missense LOH	Aavikko et al., 2012
	Gli3	7p14.1	Pancreatic cancer	Somatic	Missense	Jones et al., 2008
			Leukemia	Germline Somatic	Missense	Dagklis et al., 2015; Burns et al., 2018
			Colorectal cancer	NA	Missense	Wood et al., 2007
Gain of function	Gli1	12q13.3	Rhabdomyosarcoma	Germline	Amplification	Roberts et al., 1989
			Medulloblastoma	Somatic	Amplification	Thompson et al., 2006; Northcott et al., 2009
			Leukemia	Somatic	Missense	Dagklis et al., 2015; Burns et al., 2018
			Breast cancer	Somatic	Amplification	Nessling et al., 2005; Wood et al., 2007; Jiao et al., 2012
			Pancreatic cancer	Somatic	Missense	Jones et al., 2008
			Colorectal cancer	Somatic	Frameshift	Lee et al., 2018
	Gli2	2q14.2	Medulloblastoma	Somatic	Amplification	Northcott et al., 2009; Rusert et al., 2014; Kool et al., 2014
			Leukemia	Somatic	Missense	Burns et al., 2018;
	SMO	7q32.1	Medulloblastoma	Somatic	Missense	Reifenberger et al., 1998; Rusert et al., 2014; Kool et al., 2014
			Basal cell carcinoma	Somatic	Missense	Reifenberger et al., 1998; Reifenberger et al., 2005
			Leukemia	Somatic	Frameshift	Dagklis et al., 2015
			Gastric–intestinal cancer	NA	Missense Insertion	Wang et al., 2013
			Ameloblastoma	Somatic	Missense	Sweeney et al., 2014
	SHH	7q36.3	Medulloblastoma	NA	Amplification	Reifenberger et al., 1998; Kool et al., 2014
			Basal cell carcinoma	Somatic	Missense	Couve-Privat et al., 2004

Notably, it is known that patients with Gorlin syndrome (GS), also called nevoid basal cell carcinoma syndrome (nevoid BCC) or basal cell nevus syndrome, are predisposed to multiple cancers, including basal cell carcinoma (BCC), medulloblastoma (MB), and rhabdomyosarcoma (Johnson et al., 1996; Jones et al., 2011). Gorlin syndrome patients carry mutations in *Ptch1* and *SuFu* genes (Hahn et al., 1996; Smith et al., 2014). Of note, *SuFu* germline mutations are present with a low frequency (Smith et al., 2014). By contrast, heterozygous germline mutations in *Ptch1*, including nonsense, missense, splicing mutations, as well as loss of heterozygosity (LOH) (Burns et al., 2018), are typical alterations in these patients, inducing different tumors (Lindstrom et al., 2006). In patients with GS, the loss of function of the *Ptch1* gene permits SMO to move into the PC, resulting in an aberrant activation of Hh signaling, which drives cellular growth of these tumors. Notably, the *Ptch1* knock-out mouse model develops tumors similar to Gorlin syndrome patients, like BCC, MBs, and sporadic rhabdomyosarcomas, providing evidence about the driver role of Hh signaling for cancer development (Hahn et al., 1996; Nitzki et al., 2011). The co-presence of *Ptch1* and *SuFu* mutations or *Gli1* amplifications is also identified in rhabdomyosarcoma patients (Bridge et al., 2000; Roberts et al., 1989). Finally, combined heterozygous *Ptch1* and *Ptch2* germline mutations are observed in newborns with rhabdomyosarcoma (Taeubner et al., 2018), suggesting that haploinsufficiency of *Ptch* is sufficient to sustain tumor development in the absence of LOH (Nilsson et al., 2000). In addition, MB is one the most common brain tumors affecting mostly children and is a typical GS-related cancer (Jones et al., 2011; Huttner, 2012). GS patients characterized by *SuFu* mutations display a higher risk of developing MB than *Ptch1*-mutated patients. Based on the molecular features that are involved in MB pathogenesis, it is possible to distinguish four groups: SHh-activated, WNT-activated, c-Myc–activated, and Group 4 associated with isochromosome 17q (Kool et al., 2012; Rusert et al., 2014). In MB, Hh-dependent tumorigenesis may depend on an aberrant regulation of Hh ligands or a deregulation of Hh signaling with an altered expression/function of downstream components, such as loss-of-function mutations of *Ptch1* or *SUFU* (Taylor et al., 2002; Kool et al., 2012) or, conversely, activating mutations of *SMO* and *SHh* (Reifenberger et al., 1998; Zurawel et al., 2000). Of note, somatic copy number variations of *Gli1* and *Gli2* genes are found in this subgroup (Thompson et al., 2006; Northcott et al., 2009). All these mutations allow ligand-independent Hh signaling to promote cell growth and increase tumorigenesis.

Alterations in Hh signaling are also described in meningiomas, the most common primary tumor of the central nervous system (Wiemels et al., 2010). Aavikko and colleagues identified a germline *SuFu* mutation as the cause of multiple meningiomas in a single large Finnish family, suggesting that *SuFu* alterations predispose to meningiomas in addition to MBs (Aavikko et al., 2012).

BCC is a common type of skin cancer, representing almost 80% of non-melanoma skin cancer (NMSC), with an incidence that increases by 10% per year (Bakshi et al., 2017). As argued above, BCC is linked to GS and is characterized by germline mutations of *Ptch1* and *SuFu*. Accordingly, sporadic BCC presents hyperactivated Hh signaling, associated with inactivating mutations in *Ptch1* and activating mutations in *SMO*, with a high-rate frequency (Gailani et al., 1996; Reifenberger et al., 1998; Xie et al., 1998). Moreover, loss of *SuFu* function is also found in sporadic BCC (Reifenberger et al., 2005). Couvè-Privat and colleagues identify the presence of ultraviolet (UV)-specific mutations in the NH2-terminal domain of SHh in BCC patients with xeroderma pigmentosum (Couve-Privat et al., 2004). The current opinion is that during BCC genesis, SHh signaling aberrantly activated is necessary, maybe sufficient, to develop the malignancy (Teglund and Toftgard, 2010). In addition, there are numerous types of cancer associated with Hh signaling activation. Genetic aberrations on Hh signaling are found also in a subset of breast cancer, characterized by loss of function in *Ptch1* and gain of function of *Gli1* (Naylor et al., 2005; Nessling et al., 2005; Wood et al., 2007; Sinha et al., 2008; Jiao et al., 2012). A global genomic analysis from twenty-four advanced pancreatic adenocarcinomas highlighted the presence of missense mutations in *Gli1* and *Gli3* (Jones et al., 2008), and Hh signaling pathway is recognized as an early and late mediator of pancreatic cancer tumorigenesis (Thayer et al., 2003). Also, in prostate cancer is identified a *SuFu* mutation (Sheng et al., 2004). Recently, in patients with T-cell acute lymphoblastic leukemia (T-ALL), several mutations of SHh signaling are described (Dagklis et al., 2016), and loss of *Ptch1* function is able to empower T-ALL development Notch1-dependent, demonstrating that Hh pathway activation is an oncogenic driver in the molecular pathogenesis of T-ALL (Burns et al., 2018). In addition, *SMO* and *Ptch1* mutations are also found in gastrointestinal tumors (Guleng et al., 2006; Wang et al., 2013). Lee and colleagues performed a single-strand conformation polymorphism analysis and DNA sequencing in samples of colorectal and gastric cancers and identified frameshift mutations of *Gli1*, associated with a microsatellite instability (MSI) phenotype. (Lee et al., 2018). Finally, *SMO* activating mutations are involved in ameloblastomas arising in the maxilla (Sweeney et al., 2014).

Although tumors not GS-related present direct mutations in genes involved in the Hh pathway, it is not possible to identify Hh signaling as the driving force for cancer onset. In fact, activated Hh signaling is able to empower the severity of the tumors, but this pathway alone is not capable of driving cancer development. A major elucidation of the mechanisms leading to the genesis of malignancy through an aberrant activation of this pathway and/or unbalanced cross-talking with other signaling pathways could provide additional therapeutic options to limit tumor growth and relapse and to reduce drug resistance.

## Communication Among Hh and Different Signaling Pathways

### Crosstalk Between Hh and Notch Signaling Pathways

The family of Notch receptors includes four heterodimeric transmembrane proteins (Notch1–4), which are activated upon interaction with different types of ligands [Serrate-like Jagged1 and 2 (JAG1–2) and Delta-like (Delta1, 3, and 4)], expressed by adjacent cells. The activation of Notch signaling requires the binding *in trans* between Notch receptors, expressed on the surface of signal-receiving cells, and Notch ligands, expressed on the surface of adjacent signal-sending cells. Such an interaction renders Notch susceptible to two sequential proteolytic cleavages that involve two distinct enzymes: an A-disintegrin and metalloprotease (ADAM) and Presenilin (PS)/γ-secretase complex. These events end in the release of a soluble Notch intracellular domain (NICD) (Mumm and Kopan, 2000; Fortini, 2009; Kopan and Ilagan, 2009; Palermo et al., 2014; Bellavia et al., 2018). In canonical Notch signaling, NICD disengages from the plasma membrane and proceeds towards the nucleus, where it associates with recombining binding protein suppressor of hairless J kappa (RBP-Jκ, also known as RBPSUH/CSL/CBF-1), a transcription repressor. CSL–NICD complex, reached by a member of Mastermind-family coactivators (MAML1–3) (Wu et al., 2000), induces the transcriptional activation of several target genes, for instance, *HES1/5*, *HEY* (Bellavia et al., 2018), *Myc* (Palomero et al., 2006; Weng et al., 2006), *cyclinD* (Joshi et al., 2009; Cohen et al., 2010), *pTalpha* (Bellavia et al., 2007b), and *Jagged1* (Pelullo et al., 2014). Notch signaling activation also occurs in a non-canonical pathway. NICD can coactivate transcription by forming a complex with the lymphoid enhancer factor 1 (LEF-1) transcription factor (Ross and Kadesch, 2001) or by binding to p50 or c-Rel in the nucleus to enhance the activity of the transcription factor NF-κB (Nuclear Factor-kappa B) (Shin et al., 2006; Vacca et al., 2006; Kumar et al., 2014), and can modulate Tal-1 and/or Ikaros activity (Beverly and Capobianco, 2003; Campese et al., 2003; Talora et al., 2006; Bellavia et al., 2007a; Talora et al., 2008). Notch signaling, like the Hh pathway, is involved in the proliferation, differentiation, and survival of multiple tissues. In the hematopoietic system, Notch can increase the survival and self-renewal of hematopoietic progenitors (Varnum-Finney et al., 2000) and controls regulatory T-cell expansion/migration to peripheral lymphoid organs (Campese et al., 2009; Ferrandino et al., 2018a; Ferrandino et al., 2018b). Very intriguing is the observation that Notch1 and Notch3 receptors are associated with different steps of ongoing thymocyte development (Felli et al., 1999). This suggests that they work with distinct timing and with separate spatial expression, underlining the absence of a functional redundancy, an observation sustained also by structural differences (Bellavia et al., 2018). A hampered Notch signaling pathway takes place in several pathological events that range from neurodegenerative disorders to cancer (Joutel et al., 2000). Persistent uncontrolled Notch signaling is responsible for the onset/progression of several tumors, such as T-cell leukemia (Checquolo et al., 2010; Cialfi et al., 2013; Vargas Romero et al., 2015; Franciosa et al., 2016; Tottone et al., 2019), B-cell leukemia (Rosati et al., 2009; Arruga et al., 2014; De Falco et al., 2015), breast cancer (Rustighi et al., 2014; Diluvio et al., 2018), colorectal (Reedijk et al., 2008; Sikandar et al., 2010), ovarian cancer (Choi et al., 2008; Steg et al., 2011; McAuliffe et al., 2012), glioma (Catanzaro et al., 2017; Ceccarelli et al., 2019), and skin cancer (Cialfi et al., 2014; Palermo et al., 2014). Numerous gain-of-function mouse models show that mutations in Notch1/3 signaling are related to rare cases of human T-ALL (Bellavia et al., 2000; Allman et al., 2001). Although first associated with T-ALL, activating Notch mutations are identified in several subtypes of B-cell malignancies (Micci et al., 2007; Rosati et al., 2009; Kuang et al., 2013; Radojcic and Maillard, 2014; De Falco et al., 2018).

Studies show that Notch and Hh signaling pathways share the exact spatio-temporal window during T-cell development even if they play a non-redundant role in orchestrating early thymocyte differentiation and proliferation before pre-T cell Receptor (pre- TCR) signal initiation (Crompton et al., 2007; Rowbotham et al., 2007; Drakopoulou et al., 2010). These findings suggest that Hh may synergize with Notch signaling to maintain an intracellular balance through a significant integration of these signaling pathways. It is known that aberrant Hh signaling contributes to tumor cell growth and survival and cancer stem cell (CSC) maintenance in lymphomas, leukemia, multiple myeloma, and B-cell chronic lymphocytic leukemia (CLL) (Dierks et al., 2007; Lindemann, 2008; Singh et al., 2010; Decker et al., 2012; Aberger et al., 2017). To unravel the molecular mechanisms that subtend in the network of cross-talking pathways could be essential in treatment of hematological disease. Interestingly, Hh signaling is a potential therapeutic target for patients with myeloid and lymphoid leukemia, and Hh pathway inhibitors are used in many preclinical studies (Dagklis et al., 2016; Rimkus et al., 2016). Despite the importance of the Hh pathway in T-cell development and in several hematological malignancies (Mar et al., 2011), the role of the Hh pathway in T-ALL is unclear, and conflicting data are reported. A study shows that Hedgehog signaling is dispensable for T-ALL development (Gao et al., 2009), whereas other studies document T-ALL cell line sensitivity to Hh inhibitors, suggesting that this pathway may be important in T-ALL development and/or maintenance (Ji et al., 2007; Hou et al., 2014; Dagklis et al., 2016). Recently, several reports highlight activating mutations in components of the Hh signaling in T-ALL, supporting the idea that Hedgehog pathway deregulation may play a role in the onset and/or development of T-ALL. Functional analysis confirms the gain-of-function properties of two truncated SMO mutations in T-ALL (Dagklis et al., 2015). Moreover, primary T-ALL cases with high Gli mRNA levels are sensitive to Hedgehog pathway inhibition by GANT61 or vismodegib in *in vivo* xenograft models (Hou et al., 2014; Dagklis et al., 2016). Despite the numerous correlations, the existence of a direct molecular connection between Hh and Notch in T-ALL is not well documented, yet. Recently, it has been reported that loss of *Ptch1* function is able to accelerate the onset of Notch1-induced human T-ALL, demonstrating that Hh pathway mutations are driver oncogenic alterations, providing a molecular rationale for targeted therapy (Burns et al., 2018).

On the contrary, a reciprocal exclusion between Hh and Notch signaling pathways is already defined in the skin. In particular, Notch1 deficiency results in an increased expression of Gli2, along with tumor development in squamous cell carcinoma (Okuyama et al., 2008).

**Figure 2:**
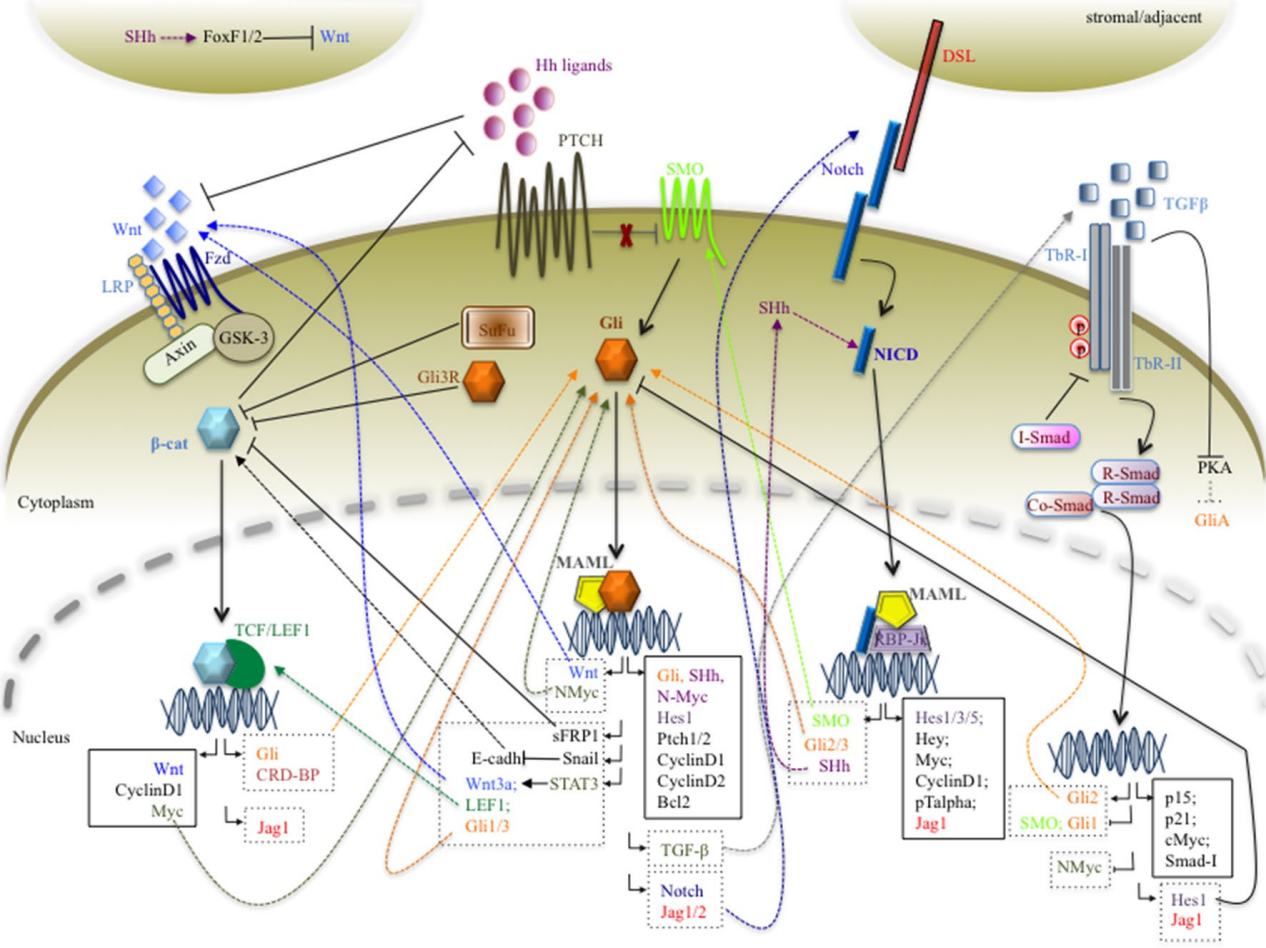
A schematic picture of the interlinking networks between Hh, Wnt/β-catenin, Notch, and TGF-β pathways inside a cancer cell. In the picture are represented the signals deriving from major signaling pathways that converge on the Hh signaling and *vice versa*. The figure is largely discussed in the main text. The black arrows represent the main signaling pathways; the dashed and truncated arrows indicate the positive and negative crosstalks between the pathways. The continuous and dashed rectangles contain canonical and non-canonical target genes, respectively.

Likewise, an inverted gradient between Hh and Notch is observed in normal colon crypt development (Kosinski et al., 2007; Takebe et al., 2011; Geissler and Zach, 2012). Very little is known regarding the crosstalk mechanisms between Hh and Notch in colorectal cancer (CRC) tumorigenesis and progression. A paper shows an antagonistic crosstalk between Hh and Notch in order to give rise to proper intestine organogenesis in the mouse, but the molecular mechanism is unknown (Kim et al., 2011). Moreover, interaction between Notch and Hedgehog signaling pathways is critical in regulating self-renewal, proliferation, and differentiation of target cells, ensuring correct organogenesis. Numerous evidence supports the significant role of Hh/Notch crosstalk in cancer biology and chemotherapy-resistant CSCs (Takebe et al., 2011). Studies suggest that crosstalk between Hh and Notch signaling pathways exists and may be involved in the regulation of embryonic stem cell fate determination (Huang et al., 2012), in self-renewal and differentiation of breast cancer cells (Guo et al., 2011), and in hepatic stellate cell fate (Xie et al., 2013). In addition, a study provides evidence that Notch signaling regulates the expression of SHh in neuronal stem cells. Notch activation leads to expression of Hes3 and SHh through activation of serine/threonine protein kinase B (Akt), signal transducer and activator of transcription 3 (STAT3), and mammalian target of rapamycin (mTOR), which promote cell survival (Androutsellis-Theotokis et al., 2006). Conversely, Gli1 is able to sustain the transcription of Jagged1 and Notch1 in the brain (Stecca and Ruiz i Altaba, 2009), and Notch ligand Jagged2 (Katoh and Katoh, 2009). On the other hand, the Notch target Hes1, a repressive transcription factor, is able to suppress Hh signaling, by inhibiting Gli1 transcription in glioma and MB tumors (Schreck et al., 2010). Of note is the observation that Hes1, the principal effector of the Notch pathway, is also a target of SHh in both in mesodermal and neural cells (Ingram et al., 2008). These data agree with the observation that in a subset of MB patients, Hes1 is upregulated and its expression correlates with shorter patient survival (Fan et al., 2004). Combined activation of Hh and Notch signaling pathways is observed in prostate cancer cells (Domingo-Domenech et al., 2012) and in the pathogenesis of pituitary adenomas (Yavropoulou et al., 2015). Furthermore, novel mechanistic insights demonstrate that Notch signaling plays a central role in left–right asymmetry, playing a crucial role in regulating cilium length (Krebs et al., 2003, Lopes et al., 2010). Interestingly, Notch signaling controls trafficking of Hh signaling mediators into the PC, sustaining the responsiveness to SHh (Kong et al., 2015, Stasiulewicz et al., 2015). Consistent with this observation, Notch and Presenilin 2, a subunit of the γ-secretase complex, localize around the PC. Notch signaling is activated by presenilin/γ-secretase activity, and the Notch intracellular domain is able to move into the nucleus to activate the transcription of several genes involved in cilium length and Hh signaling (Ezratty et al., 2011, Stasiulewicz et al., 2015). This phenomenon could be explained by the observation that the NICD/RBP-Jκ complex is able to drive the transcriptional activation of SMO and Gli2/3 in neuronal cells (Li et al., 2012) (**Figure 2**).

However, the existence of a regulatory mechanism impinging on the crosstalk between Hh and Notch in controlling tumor onset and progression needs to be better understood.

### Crosstalk Between Hh and Wnt/β-Catenin Signaling Pathways

The Wnt family of secreted glycoproteins governs multiple developmental events during embryogenesis *via* the transcriptional coactivator β-catenin, and it is also implicated in adult tissue homeostasis and repair (Logan and Nusse, 2004).

In the absence of Wnt proteins, the cytoplasmic β-catenin protein is constantly degraded by the action of a β-catenin degradation complex, composed of the tumor suppressor adenomatous polyposis coli gene product (APC) (MacDonald et al., 2009), the scaffolding protein Axin, CK1, and GSK3. Together, these proteins induce post-translational modifications, resulting in a phosphorylated β-catenin, which is recognized by β-TrCP, targeted for ubiquitination and degraded by the proteasome (He et al., 2004). Altogether, these events prevent a β-catenin shift into the nucleus, and Wnt/β-catenin target genes are thereby repressed by the T-cell factor (TCF)/LEF family of proteins (Brannon et al., 1997), associated with the transcriptional repressor Groucho/Transducin-like enhancer protein (TLE) (Cavallo et al., 1998). The Wnt/β-catenin pathway is activated when a Wnt ligand binds to the seven-pass transmembrane Frizzled (Fz or Fzd) receptor, linked to (Brannon et al., 1997) its coreceptor, the low-density lipoprotein receptor-related protein (LRP). Upon the employment of the scaffolding protein Dishevelled (Dvl), the Wnt–Fz–LRP complex induces the recruitment of the Axin complex to the receptors. These events lead to inhibition of Axin-mediated β-catenin phosphorylation and thereby to the stabilization of β-catenin, which accumulates and moves to the nucleus. Once there, β-catenin converts the TCF/Groucho/TLE repressor complex into a transcriptional activator complex that activates the expression of Wnt target genes, such as Wnt components (MacDonald et al., 2009), c-Myc (He et al., 1998), Cyclin D1 (Tetsu and McCormick, 1999), and the Notch ligand Jagged1 (Rodilla et al., 2009) (**Figure 2**). Oncogenic mutations in canonical Wnt signaling determine a constitutive activation of the pathway in a ligand-independent manner, which is linked to human congenital disorders, cancers, and other diseases (Clevers, 2006). Moreover, a recent review summarizes the wide range of epigenetic modifications of the Wnt signaling pathway that affects the development of a variety of tissues and organs, producing dramatic phenotypes and sustaining tumor formation (Wils and Bijlsma, 2018).

Recent studies show that Hh and Wnt signaling pathways can regulate each other, affecting their transcriptional output in specific cellular contexts in which they normally operate (**Figure 2**). Firstly, a crosstalk between Hh and Wnt signaling pathways is described to be involved in the onset/progression of BCC. In fact, aberrant Hh signaling activation, the key step in the tumorigenic program leading to BCC (Nilsson et al., 2000), determines the transcriptional activation of *Wnt* genes through Gli transcription factors (Bonifas et al., 2001; Katoh and Katoh, 2009). Moreover, the existence of a positive feedback loop between Hh and Wnt is present during epithelial transformation, where Gli1 activates Snail, which in turn maintains Gli1 expression through Wnt/β-catenin signaling (Li et al., 2007).

Several reports demonstrate the existence of two-way communication between Hh and Wnt. On one hand, β-catenin is able to directly enhance the luciferase activity of Gli-responsive elements (Maeda et al., 2006) and to induce the post-transcriptional stabilization of Gli mRNAs, by upregulating CRD-BP, (coding region determinant-binding protein), an RNA-binding protein (Noubissi et al., 2009). On the other hand, Gli1 is able to induce the activation of Wnt2b, Wnt4, and Wnt7b, which in turn trigger Wnt/β-catenin signaling by promoting the stability of β-catenin itself (Li et al., 2007). In keeping with these experimental data, the inhibition of Hh signaling by cyclopamine reduces β-catenin/TCF transcriptional activity, induces E-cadherin expression, and reduces invasion in CRC (Qualtrough et al., 2015). Moreover, inhibition of SHh signaling causes a reduction in Wnt-mediated transcriptional activity mediated by Gli3R, able to block the active form of the Wnt transcriptional effector, β-catenin, by physically interacting with the carboxy-terminal domain of β-catenin, a region that includes the transactivation domain (Ulloa et al., 2007).

Conversely, several data suggest an antagonism between Hh and WNT in CRC. In fact, a CRC cell line exposure to recombinant SHh results in the nuclear exit and membrane accumulation of β-catenin, consistent with its role in forming adherens junctions (Yoshimoto et al., 2012) and controlling epithelial–mesenchymal transition (EMT) (Varnat et al., 2010). In addition, it has been shown that Gli1/2 regulates the expression of secreted Frizzled-related protein sFRP-1 with a subsequent cytoplasmic accumulation of Wnt/β-catenin (He et al., 2006). All these data agree with a clinical study that demonstrated the reverse association of Gli1 and β-catenin in human samples of CRC. This study also showed that the overexpression of exogenous Gli1 determines a reduction of nuclear β-catenin in CRC cell lines (Akiyoshi et al., 2006).

Moreover, it reported that IHh, required for normal colonic epithelial differentiation (Ramalho-Santos et al., 2000), is also able to antagonize the proliferative Wnt signaling in crypts by abrogating endogenous β-catenin/TCF signaling (van den Brink et al., 2004). Consistently, an analysis of patients with APC mutations showed the loss of IHh expression in dysplastic epithelial cells present in adenomas, suggesting that IHh expression is downregulated in response to constitutive β-catenin/TCF signaling (Fu et al., 2014). In addition, Gli1 upregulates SHh expression, which is secreted from and acts on stromal cells, able to respond to SHh, enhancing Foxf1 and Foxf2 expression, which inhibit mesenchymal expression of Wnt5a and lead to suppression of β-catenin (Ormestad et al., 2006).

A controversial communication between Hh and Wnt signaling is highlighted in MB. In fact, on one hand, it is reported that SuFu is able to bind β-catenin and to export it from the nucleus and thereby to repress β-catenin/TCF–mediated transcription. Loss of SuFu function not only is associated with an increased risk of MB but also results in over-activity of both SHh and Wnt signaling pathways (Taylor et al., 2004). On the other hand, the activation of Wnt/β-catenin signaling specifically decreases the proliferation of SHh-dependent cerebellar granule cell precursors (GCPs) (Poschl et al., 2014) and of SHh-MB cells, by inducing Gli to proteosomal degradation upon a direct interaction between β-catenin and Gli proteins (Zinke et al., 2015).

Of interest is the demonstration that the mutations in Wnt signaling are recently described in about 15% of MB, corresponding to a minority, and defining a subset of patients with improved outcomes (Swartling et al., 2010). Nevertheless, N-Myc, a known target gene of the Wnt pathway, is also related to a subtype group of MB (Kool et al., 2008), suggesting its important role in the MB pathogenesis. Moreover, several groups report that SHh promotes the expression and post-transcriptional stabilization of N-Myc in mice (Kenney et al., 2003, Thomas et al., 2009).

To date, loss-of-function and gain-of-function mutations in known regulators of the Hh signaling pathway have been elucidated in hematological malignancies (Campbell and Copland, 2015), and the involvement of an autocrine and/or paracrine Hh signaling has been also demonstrated in multiple myeloma (Blotta et al., 2012), lymphoma (Dierks et al., 2007), and colon cancers (Bian et al., 2007; Yoshikawa et al., 2009). Genetic and epigenetic mutations in Wnt signaling components are also identified in leukemia (Staal et al., 2016). Intriguingly, the ability of Hh to crosstalk with Wnt is suggested by Sengupta and coworkers, who demonstrated that Hh signaling *via* Stat3 activation gives rise to the expression of Wnt3a, Lef1, Gli1–3, and other target genes in the chronic phase of chronic myeloid leukemia (CML) (Sengupta et al., 2007). Although it has been reported that the Hh inhibitor cyclopamine and the WNT inhibitor quercitin suppress the growth of leukemia cells (Kawahara et al., 2009), direct crosstalk between Hedgehog and Wnt/β-catenin in hematological disorders has not yet been well evaluated (**Figure 2**).

### Crosstalk Between Hh and TGF-β Signaling Pathways

Mammalian TGF-β family members include more than 35 structurally related secreted proteins, including TGF-βs *stricto sensu*, bone morphogenetic proteins (BMPs), growth differentiation factors (GDFs), glial-derived neurotrophic factors (GDNFs), Nodal, Lefty, and the Müllerian inhibitory substance/anti-Müllerian hormone (MIS/AMH) (Zi et al., 2012). Members of the TGF-β family play fundamental roles during embryonic development and in maintenance of tissue homeostasis since they regulate diverse cellular processes, such as proliferation, differentiation, migration, and extracellular matrix synthesis (Verrecchia and Mauviel, 2007).

Family members of TGF-β *stricto sensu* are identified in three isoforms: TGF-β1, TGF-β2, and TGF-β3. They signal *via* cell-surface receptors, which consist of specific hetero-oligomeric complex of type-I and type-II serine/threonine kinase receptors (TbR-I and TbR-II). Typically, TGF-β signaling initiates with the binding to and subsequently activation of TbR-I and TbR-II. Smad proteins are the initial responders and transduce the signal from the TGF-β receptor activation process. Smads comprise a family of structurally similar proteins with different functions: receptor-regulated (R-Smads), common mediator (Co-Smads), and inhibitory (I-Smads). After ligand binding, the TbR-II receptor phosphorylates TbR-I, which in turn phosphorylates and activates R-Smads (e.g., Smad2 and Smad3). Then, the R-Smads bind to Co-Smads (e.g., Smad4), and this complex translocates to the nucleus, where it regulates the transcription of TGF-β–responsive genes, involved in cytostatic and/or apoptotic events (p15 and p21) (Pardali et al., 2000; Seoane et al., 2001; Siegel and Massague, 2003), proliferation (c-Myc) (Chen et al., 2002), and the transcription of elements of other signaling pathways, such as Hes1 and Jagged1 (Zavadil et al., 2004). On the contrary, the I-Smads exert a negative feedback effect by competing with R-Smads for interaction with the receptor. Therefore, the I-Smads (e.g., Smad7) associate with ligand–TbR-I receptor complex and interfere with phosphorylation of R-Smads (e.g., Smad3), by preventing their interaction with activated TbR-I. Since the expression of Smad7 is induced by TGF-β1, in turn, it inhibits TGF-β signaling by a negative feedback system (Wahl, 1994, Moustakas et al., 2001). Several publications display a crosstalk between TGF-β and Hh signaling pathways in cancer (**Figure 2**). In the onset of BCC, GLI2 is identified as an early target gene of the TGF-β/SMAD cascade, independently by Hedgehog signaling activity (Dennler et al., 2007). Interestingly, Gli1 is induced by TGF-β in a Gli2-dependent manner, and such an induction is not inhibited by cyclopamine, an SMO antagonist, demonstrating that Hh signaling is not required (Dennler et al., 2007). Concomitantly, studies show that TGF-β inhibits PKA activity, which may contribute to increasing the pool of Gli activator proteins (Pierrat et al., 2012). In addition, Hh signaling induced the expression of TGF-β members. In detail, in the mouse model of SMO-mediated BCC, Fan and colleagues identify TGF-β2 as an Hh target gene (Fan et al., 2010). Thus, the crosstalk between Hh and TGF-β may activate a vicious circuitry, able to promote and amplify downstream targets of Gli1/2, which in turn sustains the activation of TGF-β and Hh itself (**Figure 2**).

During the development of the cerebellum, the GCPs express BMPs. It is reported that in GCPs, BMP‐2 and ‐4 antagonize the proliferative effects of Hh through SMAD5. In addition, BMPs downregulate Hh signaling proteins such as SMO and Gli1. Moreover, it is suggested that BMP‐2 inhibits proliferation and promotes cell differentiation of GCPs, by downregulating the oncogene N-Myc (Alvarez-Rodriguez et al., 2007).

It is well described that the architectural structure of the colon is controlled by a gradient of WNT, Hh, BMP, and Notch signaling (Geissler and Zach, 2012). In particular, BMP signaling exerts its function in the differentiated compartment at the top of the crypt, while it is relatively inactive in early compartments at the bottom of the crypt, due to the presence of the BMP inhibitor, Noggin (Syed, 2016). Thus, the Hh and BMP overlapping signaling pathways in the crypt regulate stem cell self-renewal, proliferation, and differentiation, but the existence of a direct regulation between them is not defined yet. However, loss of phosphorylation of Smad1, Smad5, and Smad8 is observed in 70% of sporadic colon cancer (Kodach et al., 2008). Loss of Smad4 or BMPRII function is the likely mechanistic basis for inactivated BMP signaling in sporadic colon cancer. In fact, several reports support the hypothesis that TGF-β/BMP signaling is involved in invasion/metastasis events in tumor cells (Zavadil and Bottinger, 2005).

Although TGF-β is proposed to be a potent negative regulator of hematopoiesis (Fortunel et al., 2000; Fortunel et al., 2003) and the loss of TGF-β signaling is reported in several leukemias (Le Bousse-Kerdiles et al., 1996; DeCoteau et al., 1997; Jakubowiak et al., 2000; Imai et al., 2001; Wolfraim et al., 2004), the disruption of TGF-β signaling alone is not sufficient to induce leukemia (Datto et al., 1999, Yang et al., 1999; Larsson et al., 2003). Thus, its role in leukemogenesis and its ability to crosstalk with other signaling pathways remain largely unknown.

## Conclusion

Biological processes such as stem cell self-renewal, cell growth, and differentiation are orchestrated by a number of major signaling pathways that integrate with each other to modulate cell outcomes in response to several intracellular signals.

The picture in **Figure 2** schematically summarizes the intricate molecular crosstalk between Hh, Notch, Wnt, and TGF-β, which establishes a network of protein–protein interactions able to affect gene expression programs across the pathways. The cartoon clearly represents the complexity of molecular interlinking inside the tumor cell, which is underlying the severity of the neoplasia phenomenon. The pathogenesis of malignancies is characterized by dysregulation of at least two pathways at the same time, proving a functional advantage to neoplastic cells, influencing relapse and drug responsiveness. We need to unravel the intricate signaling networks between the major pathways to prevent tumor formation and/or to contribute to the development of novel anticancer treatment modalities. Aberrant activation of the Hh pathway is tightly associated with cancer development. Notably, a plethora of stimuli activate multiple signaling cascades in tumor cells, which can impinge on Hh signaling and affect its output, by direct protein–protein interaction or by regulating indirectly the expression of key components of the signaling. Conversely, the Hh pathway can also have an impact on other pathways, determining a mutual signaling interference, a phenomenon underlying an unbalanced network in cancer cells. The Hh signaling pathway is considered an important molecular cross point able to integrate/synergize with other major signaling cascades, such as Wnt, TGF-β, and Notch. Therefore, **Figure 2** presents an overview concerning direct or indirect molecular mechanisms that sustain Hh cross-talking with major signaling pathways. Molecular sharing between Hh and the Wnt/β-catenin, Notch, or TGF-β signaling pathway suggests that two major signaling networks crosstalk with each other during oncogenesis. Therefore, a combined involvement of these signaling pathways in early stages of tumorigenesis as well as in the metastatic process in several types of cancers suggests the need to combine novel targeted inhibitors with standard antineoplastic therapy to enhance therapy efficacy.

This complex scenario could open a huge window of opportunity for the development of new therapeutic drugs for multiple cancers.

## Author Contributions

MP, SZ, and DB wrote the paper. MP, SZ, and FN prepared the figures. SC, IS, and DB reviewed drafts of the paper. All authors read and approved the final manuscript.

## Funding

This work was supported by the following grants: Sapienza University (Progetti H2020 collaborativi) #PH118164340087CF (to IS) and Sapienza University 2016 project #RP116154CE3DD739 (to DB).

## Conflict of Interest Statement

The authors declare that the research was conducted in the absence of any commercial or financial relationships that could be construed as a potential conflict of interest.

## References

[B1] Aavikko M. Li S. P. Saarinen S. Alhopuro P. Kaasinen E. Morgunova E. (2012). Loss of SUFU function in familial multiple meningioma. Am. J. Hum. Genet. 91, 520–526. 10.1016/j.ajhg.2012.07.015 22958902 PMC3511996

[B2] Aberger F. Hutterer E. Sternberg C. Del Burgo P. J. Hartmann T. N. (2017). Acute myeloid leukemia—strategies and challenges for targeting oncogenic Hedgehog/GLI signaling. Cell Commun. Signal 15, 8. 10.1186/s12964-017-0163-4 28122581 PMC5267446

[B3] Akiyoshi T. Nakamura M. Koga K. Nakashima H. Yao T. Tsuneyoshi M. (2006). Gli1, downregulated in colorectal cancers, inhibits proliferation of colon cancer cells involving Wnt signalling activation. Gut 55, 991–999. 10.1136/gut.2005.080333 16299030 PMC1856354

[B4] Allman D. Karnell F. G. Punt J. A. Bakkour S. Xu L. Myung P. (2001). Separation of Notch1 promoted lineage commitment and expansion/transformation in developing T cells. J. Exp. Med. 194, 99–106. 10.1084/jem.194.1.99 11435476 PMC2193437

[B5] Alvarez-Rodriguez R. Barzi M. Berenguer J. Pons S. (2007). Bone morphogenetic protein 2 opposes Shh-mediated proliferation in cerebellar granule cells through a TIEG-1-based regulation of Nmyc. J. Biol. Chem. 282, 37170–37180. 10.1074/jbc.M705414200 17951258

[B6] Androutsellis-Theotokis A. Leker R. R. Soldner F. Hoeppner D. J. Ravin R. Poser S. W. (2006). Notch signalling regulates stem cell numbers *in vitro* and *in vivo*. Nature 442, 823–826. 10.1038/nature04940 16799564

[B7] Arruga F. Gizdic B. Serra S. Vaisitti T. Ciardullo C. Coscia M. (2014). Functional impact of NOTCH1 mutations in chronic lymphocytic leukemia. Leukemia 28, 1060–1070. 10.1038/leu.2013.319 24170027

[B8] Bai C. B. Stephen D. Joyner A. L. (2004). All mouse ventral spinal cord patterning by hedgehog is Gli dependent and involves an activator function of Gli3. Dev. Cell 6, 103–115. 10.1016/S1534-5807(03)00394-0 14723851

[B9] Bakshi A. Chaudhary S. C. Rana M. Elmets C. A. Athar M. (2017). Basal cell carcinoma pathogenesis and therapy involving hedgehog signaling and beyond. Mol. Carcinog. 56, 2543–2557. 10.1002/mc.22690 28574612 PMC5962346

[B10] Bellavia D. Campese A. F. Alesse E. Vacca A. Felli M. P. Balestri A. (2000). Constitutive activation of NF-kappaB and T-cell leukemia/lymphoma in Notch3 transgenic mice. EMBO J. 19, 3337–3348. 10.1093/emboj/19.13.3337 10880446 PMC313949

[B11] Bellavia D. Mecarozzi M. Campese A. F. Grazioli P. Gulino A. Screpanti I. (2007a). Notch and Ikaros: not only converging players in T cell leukemia. Cell Cycle 6, 2730–2734. 10.4161/cc.6.22.4894 18032925

[B12] Bellavia D. Mecarozzi M. Campese A. F. Grazioli P. Talora C. Frati L., (2007b). Notch3 and the Notch3-upregulated RNA-binding protein HuD regulate Ikaros alternative splicing. EMBO J. 26, 1670–1680. 10.1038/sj.emboj.7601626 17332745 PMC1829386

[B13] Bellavia D. Palermo R. Felli M. P. Screpanti I. Checquolo S. (2018). Notch signaling as a therapeutic target for acute lymphoblastic leukemia. Expert Opin. Ther. Targets 22, 331–342. 10.1080/14728222.2018.1451840 29527929

[B14] Beverly L. J. Capobianco A. J. (2003). Perturbation of Ikaros isoform selection by MLV integration is a cooperative event in Notch(IC)-induced T cell leukemogenesis. Cancer cell 3, 551–564. 10.1016/S1535-6108(03)00137-5 12842084

[B15] Bian Y. H. Huang S. H. Yang L. Ma X. L. Xie J. W. Zhang H. W. (2007). Sonic hedgehog–Gli1 pathway in colorectal adenocarcinomas. World J. Gastroenterol. 13, 1659–1665. 10.3748/wjg.v13.i11.1659 17461467 PMC4146943

[B16] Bigelow R. L. Chari N. S. Unden A. B. Spurgers K. B. Lee S. Roop D. R. (2004). Transcriptional regulation of bcl-2 mediated by the sonic hedgehog signaling pathway through gli-1. J. Biol. Chem. 279, 1197–1205. 10.1074/jbc.M310589200 14555646

[B17] Blotta S. Jakubikova J. Calimeri T. Roccaro A. M. Amodio N. Azab A. K. (2012). Canonical and noncanonical Hedgehog pathway in the pathogenesis of multiple myeloma. Blood 120, 5002–5013. 10.1182/blood-2011-07-368142 22821765 PMC3525024

[B18] Bonifas J. M. Pennypacker S. Chuang P. T. McMahon A. P. Williams M. Rosenthal A. (2001). Activation of expression of hedgehog target genes in basal cell carcinomas. J. Investig. Dermatol. 116, 739–742. 10.1046/j.1523-1747.2001.01315.x 11348463

[B19] Brannon M. Gomperts M. Sumoy L. Moon R. T. Kimelman D. (1997). A beta-catenin/XTcf-3 complex binds to the siamois promoter to regulate dorsal axis specification in *Xenopus*. Genes Dev. 11, 2359–2370. 10.1101/gad.11.18.2359 9308964 PMC316518

[B20] Bridge J. A. Liu J. Weibolt V. Baker K. S. Perry D. Kruger R. (2000). Novel genomic imbalances in embryonal rhabdomyosarcoma revealed by comparative genomic hybridization and fluorescence *in situ* hybridization: an intergroup rhabdomyosarcoma study. Genes Chromosomes Cancer 27, 337–344. 10.1002/(SICI)1098-2264(200004)27:4<337::AID-GCC1>3.0.CO;2-1 10719362

[B21] Burns M. A. Liao Z. W. Yamagata N. Pouliot G. P. Stevenson K. E. Neuberg D. S. (2018). Hedgehog pathway mutations drive oncogenic transformation in high-risk T-cell acute lymphoblastic leukemia. Leukemia 32, 2126–2137. 10.1038/s41375-018-0097-x 29654263 PMC6148437

[B22] Campbell V. Copland M. (2015). Hedgehog signaling in cancer stem cells: a focus on hematological cancers. Stem Cells Cloning 8, 27–38. 10.2147/SCCAA.S58613 25691811 PMC4325629

[B23] Campese A. F. Bellavia D. Gulino A. Screpanti I. (2003). Notch signalling at the crossroads of T cell development and leukemogenesis. Semin. Cell Dev. Biol. 14, 151–157. 10.1016/S1084-9521(02)00184-2 12651099

[B24] Campese A. F. Grazioli P. Colantoni S. Anastasi E. Mecarozzi M. Checquolo S. (2009). Notch3 and pTalpha/pre-TCR sustain the *in vivo* function of naturally occurring regulatory T cells. Int. Immunol. 21, 727–743. 10.1093/intimm/dxp042 19461123

[B25] Canettieri G. Di Marcotullio L. Greco A. Coni S. Antonucci L. Infante P. (2010). Histone deacetylase and Cullin3–REN(KCTD11) ubiquitin ligase interplay regulates Hedgehog signalling through Gli acetylation. Nat. Cell Biol. 12, 132–142. 10.1038/ncb2013 20081843

[B26] Carballo G. B. Honorato J. R. de Lopes G. P. F. Spohr T. (2018). A highlight on Sonic hedgehog pathway. Cell Commun. Signal 16, 11. 10.1186/s12964-018-0220-7 29558958 PMC5861627

[B27] Catanzaro G. Sabato C. Russo M. Rosa A. Abballe L. Besharat Z. M. (2017). Loss of miR-107, miR-181c and miR-29a-3p promote activation of Notch2 signaling in pediatric high-grade gliomas (pHGGs). Int. J. Mol. Sci. 18, 2742–2754. 10.3390/ijms18122742 PMC5751342 29258209

[B28] Cavallo R. A. Cox R. T. Moline M. M. Roose J. Polevoy G. A. Clevers H. (1998). *Drosophila* Tcf and Groucho interact to repress Wingless signalling activity. Nature 395, 604–608. 10.1038/26982 9783586

[B29] Ceccarelli S. Megiorni F. Bellavia D. Marchese C. Screpanti I. Checquolo S. (2019). Notch3 targeting: a novel weapon against ovarian cancer stem cells. Stem Cells Int. 2019, 6264931. 10.1155/2019/6264931 30723507 PMC6339748

[B30] Checquolo S. Palermo R. Cialfi S. Ferrara G. Oliviero C. Talora C. (2010). Differential subcellular localization regulates c-Cbl E3 ligase activity upon Notch3 protein in T-cell leukemia. Oncogene 29, 1463–1474. 10.1038/onc.2009.446 19966856

[B31] Chen C. R. Kang Y. Siegel P. M. Massague J. (2002). E2F4/5 and p107 as Smad cofactors linking the TGFbeta receptor to c-myc repression. Cell 110, 19–32. 10.1016/S0092-8674(02)00801-2 12150994

[B32] Chen Y. Sasai N. Ma G. Yue T. Jia J. Briscoe J. (2011). Sonic Hedgehog dependent phosphorylation by CK1alpha and GRK2 is required for ciliary accumulation and activation of smoothened. PLoS Biol. 9, e1001083. 10.1371/journal.pbio.1001083 21695114 PMC3114773

[B33] Choi J. H. Park J. T. Davidson B. Morin P. J. Shih Ie M. Wang T. L. (2008). Jagged-1 and Notch3 juxtacrine loop regulates ovarian tumor growth and adhesion. Cancer Res. 68, 5716–5723. 10.1158/0008-5472.CAN-08-0001 18632624 PMC2562671

[B34] Cialfi S. Palermo R. Manca S. Checquolo S. Bellavia D. Pelullo M. (2013). Glucocorticoid sensitivity of T-cell lymphoblastic leukemia/lymphoma is associated with glucocorticoid receptor-mediated inhibition of Notch1 expression. Leukemia 27, 485–488. 10.1038/leu.2012.192 22846929

[B35] Cialfi S. Palermo R. Manca S. De Blasio C. Vargas Romero P. Checquolo S. (2014). Loss of Notch1-dependent p21(Waf1/Cip1) expression influences the Notch1 outcome in tumorigenesis. Cell cycle 13, 2046–2055. 10.4161/cc.29079 24801890 PMC4111696

[B36] Clevers H. (2006). Wnt/beta-catenin signaling in development and disease. Cell 127, 469–480. 10.1016/j.cell.2006.10.018 17081971

[B37] Cohen B. Shimizu M. Izrailit J. Ng N. F. Buchman Y. Pan J. G. (2010). Cyclin D1 is a direct target of JAG1-mediated Notch signaling in breast cancer. Breast Cancer Res. Treat. 123, 113–124. 10.1007/s10549-009-0621-9 19915977

[B38] Coni S. Antonucci L. D'Amico D. Di Magno L. Infante P. De Smaele E. (2013). Gli2 acetylation at lysine 757 regulates hedgehog-dependent transcriptional output by preventing its promoter occupancy. PloS One 8, e65718. 10.1371/journal.pone.0065718 23762415 PMC3675076

[B39] Couve-Privat S. Le Bret M. Traiffort E. Queille S. Coulombe J. Bouadjar B. (2004). Functional analysis of novel sonic hedgehog gene mutations identified in basal cell carcinomas from xeroderma pigmentosum patients. Cancer Res. 64, 3559–3565. 10.1158/0008-5472.CAN-03-4040 15150112

[B40] Crompton T. Outram S. V. Hager-Theodorides A. L. (2007). Sonic hedgehog signalling in T-cell development and activation. Nat. Rev. Immunol. 7, 726–735. 10.1038/nri2151 17690714

[B41] Dagklis A. Demeyer S. De Bie J. Radaelli E. Pauwels D. Degryse S. (2016). Hedgehog pathway activation in T-cell acute lymphoblastic leukemia predicts response to SMO and GLI1 inhibitors. Blood 128, 2642–2654. 10.1182/blood-2016-03-703454 27694322

[B42] Dagklis A. Pauwels D. Lahortiga I. Geerdens E. Bittoun E. Cauwelier B. (2015). Hedgehog pathway mutations in T-cell acute lymphoblastic leukemia. Haematologica 100, e102–e105. 10.3324/haematol.2014.119248 25527561 PMC4349289

[B43] Dai P. Akimaru H. Tanaka Y. Maekawa T. Nakafuku M. Ishii S. (1999). Sonic Hedgehog-induced activation of the Gli1 promoter is mediated by GLI3. J. Biol. Chem. 274, 8143–8152. 10.1074/jbc.274.12.8143 10075717

[B44] Datto M. B. Frederick J. P. Pan L. Borton A. J. Zhuang Y. Wang X. F. (1999). Targeted disruption of Smad3 reveals an essential role in transforming growth factor beta-mediated signal transduction. Mol. Cell. Biol. 19, 2495–2504. 10.1128/MCB.19.4.2495 10082515 PMC84042

[B45] De Falco F. Del Papa B. Baldoni S. Sabatini R. Falzetti F. Di Ianni M. (2018). IL-4-dependent Jagged1 expression/processing is associated with survival of chronic lymphocytic leukemia cells but not with Notch activation. Cell Death Dis 9, 1160. 10.1038/s41419-018-1185-6 30478302 PMC6255763

[B46] De Falco F. Sabatini R. Falzetti F. Di Ianni M. Sportoletti P. Baldoni S. (2015). Constitutive phosphorylation of the active Notch1 intracellular domain in chronic lymphocytic leukemia cells with NOTCH1 mutation. Leukemia 29, 994–998. 10.1038/leu.2014.329 25425197

[B47] Decker S. Zirlik K. Djebatchie L. Hartmann D. Ihorst G. Schmitt-Graeff A. (2012). Trisomy 12 and elevated GLI1 and PTCH1 transcript levels are biomarkers for Hedgehog-inhibitor responsiveness in CLL. Blood 119, 997–1007. 10.1182/blood-2011-06-359075 22130798

[B48] DeCoteau J. F. Knaus P. I. Yankelev H. Reis M. D. Lowsky R. Lodish H. F. (1997). Loss of functional cell surface transforming growth factor beta (TGF-beta) type 1 receptor correlates with insensitivity to TGF-beta in chronic lymphocytic leukemia. Proc. Natl Acad. Sci. U. S. A. 94, 5877–5881. 10.1073/pnas.94.11.5877 9159168 PMC20874

[B49] Denef N. Neubuser D. Perez L. Cohen S. M. (2000). Hedgehog induces opposite changes in turnover and subcellular localization of patched and smoothened. Cell 102, 521–531. 10.1016/S0092-8674(00)00056-8 10966113

[B50] Dennler S. Andre J. Alexaki I. Li A. Magnaldo T. Dijke P. (2007). Induction of sonic hedgehog mediators by transforming growth factor-beta: Smad3-dependent activation of Gli2 and Gli1 expression *in vitro* and *in vivo*. Cancer Res. 67, 6981–6986. 10.1158/0008-5472.CAN-07-0491 17638910

[B51] Di Marcotullio L. Ferretti E. Greco A. De Smaele E. Po A. Sico M. A. (2006). Numb is a suppressor of Hedgehog signalling and targets Gli1 for itch-dependent ubiquitination. Nat. Cell Biol. 8, 1415–1423. 10.1038/ncb1510 17115028

[B52] Di Marcotullio L. Ferretti E. Greco A. De Smaele E. Screpanti I. Gulino A. (2007). Multiple ubiquitin-dependent processing pathways regulate hedgehog/gli signaling: implications for cell development and tumorigenesis. Cell Cycle 6, 390–393. 10.4161/cc.6.4.3809 17312394

[B53] Di Marcotullio L. Greco A. Mazza D. Canettieri G. Pietrosanti L. Infante P. (2011). Numb activates the E3 ligase Itch to control Gli1 function through a novel degradation signal. Oncogene 30, 65–76. 10.1038/onc.2010.394 20818436

[B54] Dierks C. Grbic J. Zirlik K. Beigi R. Englund N. P. Guo G. R. (2007). Essential role of stromally induced hedgehog signaling in B-cell malignancies. Nat. Med. 13, 944–951. 10.1038/nm1614 17632527

[B55] Diluvio G. Del Gaudio F. Giuli M. V. Franciosa G. Giuliani E. Palermo R. (2018). NOTCH3 inactivation increases triple negative breast cancer sensitivity to gefitinib by promoting EGFR tyrosine dephosphorylation and its intracellular arrest. Oncogenesis 7, 42. 10.1038/s41389-018-0051-9 29795369 PMC5968025

[B56] Ding Q. Fukami Si Meng X. Nishizaki Y. Zhang X. Sasaki H. (1999). Mouse suppressor of fused is a negative regulator of sonic hedgehog signaling and alters the subcellular distribution of Gli1. Curr. Biol. 9, 1119–1122. 10.1016/S0960-9822(99)80482-5 10531011

[B57] Domingo-Domenech J. Vidal S. J. Rodriguez-Bravo V. Castillo-Martin M. Quinn S. A. Rodriguez-Barrueco R. (2012). Suppression of acquired docetaxel resistance in prostate cancer through depletion of notch- and hedgehog-dependent tumor-initiating cells. Cancer Cell 22, 373–388. 10.1016/j.ccr.2012.07.016 22975379 PMC5989708

[B58] Drakopoulou E. Outram S. V. Rowbotham N. J. Ross S. E. Furmanski A. L. Saldana J. I. (2010). Non-redundant role for the transcription factor Gli1 at multiple stages of thymocyte development. Cell Cycle 9, 4144–4152. 10.4161/cc.9.20.13453 20935514 PMC3055198

[B59] Dunaeva M. Michelson P. Kogerman P. Toftgard R. (2003). Characterization of the physical interaction of Gli proteins with SUFU proteins. J. Biol. Chem. 278, 5116–5122. 10.1074/jbc.M209492200 12426310

[B60] Ezratty E. J. Stokes N. Chai S. Shah A. S. Williams S. E. Fuchs E. (2011). A role for the primary cilium in Notch signaling and epidermal differentiation during skin development. Cell 145, 1129–1141. 10.1016/j.cell.2011.05.030 21703454 PMC3135909

[B61] Fan Q. He M. Sheng T. Zhang X. Sinha M. Luxon B. (2010). Requirement of TGFbeta signaling for SMO-mediated carcinogenesis. J. Biol. Chem. 285, 36570–36576. 10.1074/jbc.C110.164442 20858897 PMC2978585

[B62] Fan X. Mikolaenko I. Elhassan I. Ni X. Wang Y. Ball D. (2004). Notch1 and notch2 have opposite effects on embryonal brain tumor growth. Cancer Res. 64, 7787–7793. 10.1158/0008-5472.CAN-04-1446 15520184

[B63] Fan X. Eberhart C. G. (2008). Medulloblastoma stem cells. J. Clin. Oncol. 26 (17), 2821–2827. 10.1200/JCO.2007.15.2264 18539960 PMC4508659

[B64] Felli M. P. Maroder M. Mitsiadis T. A. Campese A. F. Bellavia D. Vacca A. (1999). Expression pattern of Notch1, 2 and 3 and Jagged1 and 2 in lymphoid and stromal thymus components: distinct ligand-receptor interactions in intrathymic T cell development. Int. Immunol. 11, 1017–1025. 10.1093/intimm/11.7.1017 10383933

[B65] Ferrandino F. Bernardini G. Tsaouli G. Grazioli P. Campese A. F. Noce C. (2018a). Intrathymic Notch3 and CXCR4 combinatorial interplay facilitates T-cell leukemia propagation. Oncogene 37, 6285–6298. 10.1038/s41388-018-0401-2 30038265 PMC6284016

[B66] Ferrandino F. Grazioli P. Bellavia D. Campese A. F. Screpanti I. Felli M. P. (2018b). Notch and NF-kappaB: coach and players of regulatory T-cell response in cancer. Front. Immunol. 9, 2165. 10.3389/fimmu.2018.02165 30364244 PMC6193072

[B67] Fortini M. E. (2009). Notch signaling: the core pathway and its posttranslational regulation. Dev. Cell 16, 633–647. 10.1016/j.devcel.2009.03.010 19460341

[B68] Fortunel N. O. Hatzfeld A. Hatzfeld J. A. (2000). Transforming growth factor-beta: pleiotropic role in the regulation of hematopoiesis. Blood 96, 2022–2036. 10979943

[B69] Fortunel N. O. Hatzfeld J. A. Monier M. N. Hatzfeld A. (2003). Control of hematopoietic stem/progenitor cell fate by transforming growth factor-beta. Oncol. Res. 13, 445–453. 10.3727/096504003108748483 12725536

[B70] Franciosa G. Diluvio G. Gaudio F. D. Giuli M. V. Palermo R. Grazioli P. (2016). Prolyl-isomerase Pin1 controls Notch3 protein expression and regulates T-ALL progression. Oncogene 35, 4741–4751. 10.1038/onc.2016.5 26876201 PMC5024153

[B71] Fu X. Shi L. Zhang W. Zhang X. Peng Y. Chen X. (2014). Expression of Indian hedgehog is negatively correlated with APC gene mutation in colorectal tumors. Int. J. Clin. Exp. Med. 7, 2150–2155. 25232400 PMC4161560

[B72] Fujii K. Ohashi H. Suzuki M. Hatsuse H. Shiohama T. Uchikawa H. (2013). Frameshift mutation in the PTCH2 gene can cause nevoid basal cell carcinoma syndrome. Fam. Cancer 12, 611–614 10.1007/s10689-013-9623-1 23479190

[B73] Gailani M. R. Stahle-Backdahl M. Leffell D. J. Glynn M. Zaphiropoulos P. G. Pressman C. (1996). The role of the human homologue of *Drosophila* patched in sporadic basal cell carcinomas. Nat. Genet. 14, 78–81. 10.1038/ng0996-78 8782823

[B74] Gao J. Graves S. Koch U. Liu S. Jankovic V. Buonamici S. (2009). Hedgehog signaling is dispensable for adult hematopoietic stem cell function. Cell Stem Cell 4, 548–558. 10.1016/j.stem.2009.03.015 19497283 PMC2914688

[B75] Geissler K. Zach O. (2012). Pathways involved in *Drosophila* and human cancer development: the Notch, Hedgehog, Wingless, Runt, and Trithorax pathway. Ann. Hematol 91, 645–669. 10.1007/s00277-012-1435-0 22418742

[B76] Goetz S. C. Anderson K. V. (2010). The primary cilium: a signalling centre during vertebrate development. Nat. Rev. Genet. 11, 331–344. 10.1038/nrg2774 20395968 PMC3121168

[B77] Guleng B. Tateishi K. Ohta M. Asaoka Y. Jazag A. Lin L. J. (2006). Smoothened gene mutations found in digestive cancer have no aberrant Hedgehog signaling activity. J. Gastroenterol. 41, 1238–1239. 10.1007/s00535-006-1955-2 17287906

[B78] Guo S. Liu M. Gonzalez-Perez R. R. (2011). Role of Notch and its oncogenic signaling crosstalk in breast cancer. Biochim. Biophys. Acta 1815, 197–213. 10.1016/j.bbcan.2010.12.002 21193018 PMC3060666

[B79] Hahn H. Wicking C. Zaphiropoulous P. G. Gailani M. R. Shanley S. Chidambaram A. (1996). Mutations of the human homolog of *Drosophila* patched in the nevoid basal cell carcinoma syndrome. Cell 85, 841–851. 10.1016/S0092-8674(00)81268-4 8681379

[B80] Harris P. J. Speranza G. Dansky Ullmann C. (2012). Targeting embryonic signaling pathways in cancer therapy. Expert Opin. Ther. Targets 16, 131–145. 10.1517/14728222.2011.645808 22239436

[B81] He J. Sheng T. Stelter A. A. Li C. Zhang X. Sinha M. (2006). Suppressing Wnt signaling by the hedgehog pathway through sFRP-1. J. Biol. Chem. 281, 35598–35602. 10.1074/jbc.C600200200 17035233

[B82] He T. C. Sparks A. B. Rago C. Hermeking H. Zawel L. da Costa L. T. (1998). Identification of c-MYC as a target of the APC pathway. Science 281, 1509–1512. 10.1126/science.281.5382.1509 9727977

[B83] He X. Semenov M. Tamai K. Zeng X. (2004). LDL receptor-related proteins 5 and 6 in Wnt/beta-catenin signaling: arrows point the way. Development 131, 1663–1677. 10.1242/dev.01117 15084453

[B84] Hou X. Chen X. Zhang P. Fan Y. Ma A. Pang T. (2014). Inhibition of hedgehog signaling by GANT58 induces apoptosis and shows synergistic antitumor activity with AKT inhibitor in acute T cell leukemia cells. Biochimie 101, 50–59. 10.1016/j.biochi.2013.12.019 24394624

[B85] Huang P. Xiong F. Megason S. G. Schier A. F. (2012). Attenuation of Notch and Hedgehog signaling is required for fate specification in the spinal cord. PLoS Genet. 8, e1002762. 10.1371/journal.pgen.1002762 22685423 PMC3369957

[B86] Humke E. W. Dorn K. V. Milenkovic L. Scott M. P. Rohatgi R. (2010). The output of Hedgehog signaling is controlled by the dynamic association between Suppressor of Fused and the Gli proteins. Genes Dev. 24, 670–682. 10.1101/gad.1902910 20360384 PMC2849124

[B87] Huttner A. (2012). Overview of primary brain tumors: pathologic classification, epidemiology, molecular biology, and prognostic markers. Hematol. Oncol. Clin. North Am. 26, 715–732. 10.1016/j.hoc.2012.05.004 22794280

[B88] Imai Y. Kurokawa M. Izutsu K. Hangaishi A. Maki K. Ogawa S. (2001). Mutations of the Smad4 gene in acute myelogenous leukemia and their functional implications in leukemogenesis. Oncogene 20, 88–96. 10.1038/sj.onc.1204057 11244507

[B89] Infante P. Alfonsi R. Ingallina C. Quaglio D. Ghirga F. D’Acquarica I. (2016). Inhibition of Hedgehog-dependent tumors and cancer stem cells by a newly identified naturally occurring chemotype. Cell Death Dis. 7, e2376. 10.1038/cddis.2016.195 27899820 PMC5059851

[B90] Infante P. Faedda R. Bernardi F. Bufalieri F. Lospinoso Severini L. Alfonsi R. (2018). Itch/beta-arrestin2–dependent non-proteolytic ubiquitylation of SuFu controls Hedgehog signalling and medulloblastoma tumorigenesis. Nat. Commun. 9, 976. 10.1038/s41467-018-03339-0 29515120 PMC5841288

[B91] Ingram W. J. McCue K. I. Tran T. H. Hallahan A. R. Wainwright B. J. (2008). Sonic Hedgehog regulates Hes1 through a novel mechanism that is independent of canonical Notch pathway signalling. Oncogene 27, 1489–1500. 10.1038/sj.onc.1210767 17873912

[B92] Jakubowiak A. Pouponnot C. Berguido F. Frank R. Mao S. Massague J. (2000). Inhibition of the transforming growth factor beta 1 signaling pathway by the AML1/ETO leukemia-associated fusion protein. J. Biol. Chem. 275, 40282–40287. 10.1074/jbc.C000485200 11032826

[B93] Ji Z. Mei F. C. Johnson B. H. Thompson E. B. Cheng X. (2007). Protein kinase A, not Epac, suppresses hedgehog activity and regulates glucocorticoid sensitivity in acute lymphoblastic leukemia cells. J. Biol. Chem. 282, 37370–37377. 10.1074/jbc.M703697200 17895245

[B94] Jiao X. Wood L. D. Lindman M. Jones S. Buckhaults P. Polyak K. (2012). Somatic mutations in the Notch, NF-KB, PIK3CA, and Hedgehog pathways in human breast cancers. Genes Chromosomes Cancer 51, 480–489. 10.1002/gcc.21935 22302350 PMC3302210

[B95] Johnson R. L. Rothman A. L. Xie J. Goodrich L. V. Bare J. W. Bonifas J. M. (1996). Human homolog of patched, a candidate gene for the basal cell nevus syndrome. Science 272, 1668–1671. 10.1126/science.272.5268.1668 8658145

[B96] Jones E. A. Sajid M. I. Shenton A. Evans D. G. (2011). Basal cell carcinomas in Gorlin syndrome: a review of 202 patients. J. Skin Cancer 2011, 217378. 10.1155/2011/217378 21152126 PMC2998699

[B97] Jones S. Zhang X. Parsons D. W. Lin J. C. Leary R. J. Angenendt P. (2008). Core signaling pathways in human pancreatic cancers revealed by global genomic analyses. Science 321, 1801–1806. 10.1126/science.1164368 18772397 PMC2848990

[B98] Joshi I. Minter L. M. Telfer J. Demarest R. M. Capobianco A. J. Aster J. C. (2009). Notch signaling mediates G1/S cell-cycle progression in T cells *via* cyclin D3 and its dependent kinases. Blood 113, 1689–1698. 10.1182/blood-2008-03-147967 19001083 PMC2647664

[B99] Joutel A. Dodick D. D. Parisi J. E. Cecillon M. Tournier-Lasserve E. Bousser M. G. (2000). De novo mutation in the Notch3 gene causing CADASIL. Ann. Neurol 47, 388–391. 10.1002/1531-8249(200003)47:3<388::AID-ANA19>3.0.CO;2-Q 10716263

[B100] Katoh Y. Katoh M. (2009). Hedgehog target genes: mechanisms of carcinogenesis induced by aberrant hedgehog signaling activation. Curr. Mol. Med. 9, 873–886. 10.2174/156652409789105570 19860666

[B101] Kawahara T. Kawaguchi-Ihara N. Okuhashi Y. Itoh M. Nara N. Tohda S. (2009). Cyclopamine and quercetin suppress the growth of leukemia and lymphoma cells. Anticancer Res. 29, 4629–4632. 20032413

[B102] Kenney A. M. Cole M. D. Rowitch D. H. (2003). Nmyc upregulation by sonic hedgehog signaling promotes proliferation in developing cerebellar granule neuron precursors. Development 130, 15–28. 10.1242/dev.00182 12441288

[B103] Kim T. H. Kim B. M. Mao J. Rowan S. Shivdasani R. A. (2011). Endodermal Hedgehog signals modulate Notch pathway activity in the developing digestive tract mesenchyme. Development 138, 3225–3233. 10.1242/dev.066233 21750033 PMC3133914

[B104] Kodach L. L. Wiercinska E. de Miranda N. F. Bleuming S. A. Musler A. R. Peppelenbosch M. P. (2008). The bone morphogenetic protein pathway is inactivated in the majority of sporadic colorectal cancers. Gastroenterology 134, 1332–1341. 10.1053/j.gastro.2008.02.059 18471510

[B105] Kong J. H. Yang L. Dessaud E. Chuang K. Moore D. M. Rohatgi R. (2015). Notch activity modulates the responsiveness of neural progenitors to sonic hedgehog signaling. Dev. Cell 33, 373–387. 10.1016/j.devcel.2015.03.005 25936505 PMC4449290

[B106] Kool M. Korshunov A. Remke M. Jones D. T. Schlanstein M. Northcott P. A. (2012). Molecular subgroups of medulloblastoma: an international meta-analysis of transcriptome, genetic aberrations, and clinical data of WNT, SHH, Group 3, and Group 4 medulloblastomas. Acta Neuropathol. 123, 473–484. 10.1007/s00401-012-0958-8 22358457 PMC3306778

[B107] Kool M. Koster J. Bunt J. Hasselt N. E. Lakeman A. van Sluis P. (2008). Integrated genomics identifies five medulloblastoma subtypes with distinct genetic profiles, pathway signatures and clinicopathological features. PloS One 3, e3088. 10.1371/journal.pone.0003088 18769486 PMC2518524

[B108] Kool M. Jones D. T. Jager N. Northcott P. A. Pugh T. J. Hovestadt V. (2014). Genome sequencing of SHH medulloblastoma predicts genotype-related response to smoothened inhibition. Cancer Cell 25 (3), 393–405. 10.1016/j.ccr.2014.02.004 24651015 PMC4493053

[B109] Kopan R. Ilagan M. X. (2009). The canonical Notch signaling pathway: unfolding the activation mechanism. Cell 137, 216–233. 10.1016/j.cell.2009.03.045 19379690 PMC2827930

[B110] Kosinski C. Li V. S. Chan A. S. Zhang J. Ho C. Tsui W. Y. (2007). Gene expression patterns of human colon tops and basal crypts and BMP antagonists as intestinal stem cell niche factors. Proc. Natl Acad. Sci. U. S. A. 104, 15418–15423. 10.1073/pnas.0707210104 17881565 PMC2000506

[B111] Krebs L. T. Iwai N. Nonaka S. Welsh I. C. Lan Y. Jiang R. (2003). Notch signaling regulates left–right asymmetry determination by inducing Nodal expression. Genes Dev. 17, 1207–1212. 10.1101/gad.1084703 12730124 PMC196059

[B112] Kuang S. Q. Fang Z. Zweidler-McKay P. A. Yang H. Wei Y. Gonzalez-Cervantes E. A. (2013). Epigenetic inactivation of Notch–Hes pathway in human B-cell acute lymphoblastic leukemia. PloS One 8, e61807. 10.1371/journal.pone.0061807 23637910 PMC3637323

[B113] Kumar V. Palermo R. Talora C. Campese A. F. Checquolo S. Bellavia D. (2014). Notch and NF-kB signaling pathways regulate miR-223/FBXW7 axis in T-cell acute lymphoblastic leukemia. Leukemia 28, 2324–2335. 10.1038/leu.2014.133 24727676

[B114] Larsson J. Blank U. Helgadottir H. Bjornsson J. M. Ehinger M. Goumans M. J. (2003). TGF-beta signaling-deficient hematopoietic stem cells have normal self-renewal and regenerative ability *in vivo* despite increased proliferative capacity *in vitro*. Blood 102, 3129–3135. 10.1182/blood-2003-04-1300 12842983

[B115] Le Bousse-Kerdiles M. C. Chevillard S. Charpentier A. Romquin N. Clay D. Smadja-Joffe F. (1996). Differential expression of transforming growth factor-beta, basic fibroblast growth factor, and their receptors in CD34+ hematopoietic progenitor cells from patients with myelofibrosis and myeloid metaplasia. Blood 88, 4534–4546. 8977245

[B116] Lee J. Platt K. A. Censullo P. Ruiz i Altaba A. (1997). Gli1 is a target of Sonic hedgehog that induces ventral neural tube development. Development 124, 2537–2552. 9216996 10.1242/dev.124.13.2537

[B117] Lee J. H. Song S. Y. Kim M. S. Yoo N. J. Lee S. H. (2018). Intratumoral heterogeneity of frameshift mutations of GLI1 encoding a Hedgehog signaling protein in colorectal cancers. Pathol. Oncol. Res. 24, 477–481. 10.1007/s12253-017-0272-9 28664474

[B118] Li X. Deng W. Lobo-Ruppert S. M. Ruppert J. M. (2007). Gli1 acts through Snail and E-cadherin to promote nuclear signaling by beta-catenin. Oncogene 26, 4489–4498. 10.1038/sj.onc.1210241 17297467 PMC2233601

[B119] Li Y. Hibbs M. A. Gard A. L. Shylo N. A. Yun K. (2012). Genome-wide analysis of N1ICD/RBPJ targets *in vivo* reveals direct transcriptional regulation of Wnt, SHH, and hippo pathway effectors by Notch1. Stem Cells 30, 741–752. 10.1002/stem.1030 22232070 PMC3734558

[B120] Lindemann R. K. (2008). Stroma-initiated hedgehog signaling takes center stage in B-cell lymphoma. Cancer Res. 68, 961–964. 10.1158/0008-5472.CAN-07-5500 18281468

[B121] Lindstrom E. Shimokawa T. Toftgard R. Zaphiropoulos P. G. (2006). PTCH mutations: distribution and analyses. Human Mutat. 27, 215–219. 10.1002/humu.20296 16419085

[B122] Liu S. Dontu G. Mantle I. D. Patel S. Ahn N. S. Jackson K. W. (2006). Hedgehog signaling and Bmi-1 regulate self-renewal of normal and malignant human mammary stem cells. Cancer Res. 66, 6063–6071. 10.1158/0008-5472.CAN-06-0054 16778178 PMC4386278

[B123] Logan C. Y. Nusse R. (2004). The Wnt signaling pathway in development and disease. Annu. Rev. Cell Dev. Biol. 20, 781–810. 10.1146/annurev.cellbio.20.010403.113126 15473860

[B124] Lopes S. S. Lourenco R. Pacheco L. Moreno N. Kreiling J. Saude L. (2010). Notch signalling regulates left–right asymmetry through ciliary length control. Development 137, 3625–3632. 10.1242/dev.054452 20876649

[B125] MacDonald B. T. Tamai K. He X. (2009). Wnt/beta-catenin signaling: components, mechanisms, and diseases. Dev. Cell 17, 9–26. 10.1016/j.devcel.2009.06.016 19619488 PMC2861485

[B126] Maeda O. Kondo M. Fujita T. Usami N. Fukui T. Shimokata K. (2006). Enhancement of GLI1-transcriptional activity by beta-catenin in human cancer cells. Oncol. Rep. 16, 91–96. 10.3892/or.16.1.91 16786128

[B127] Mar B. G. Amakye D. Aifantis I. Buonamici S. (2011). The controversial role of the Hedgehog pathway in normal and malignant hematopoiesis. Leukemia 25, 1665–1673. 10.1038/leu.2011.143 21660044 PMC4310480

[B128] Mastronardi F. G. Dimitroulakos J. Kamel-Reid S. Manoukian A. S. (2000). Co-localization of patched and activated sonic hedgehog to lysosomes in neurons. Neuroreport 11, 581–585. 10.1097/00001756-200002280-00030 10718318

[B129] McAuliffe S. M. Morgan S. L. Wyant G. A. Tran L. T. Muto K. W. Chen Y. S. (2012). Targeting Notch, a key pathway for ovarian cancer stem cells, sensitizes tumors to platinum therapy. Proc. Natl Acad. Sci. U. S. A. 109, E2939–E2948. 10.1073/pnas.1206400109 23019585 PMC3491453

[B130] Merchant A. A. Matsui W. (2010). Targeting Hedgehog—a cancer stem cell pathway. Clin. Cancer Res. 16, 3130–3140. 10.1158/1078-0432.CCR-09-2846 20530699 PMC2888641

[B131] Micci F. Panagopoulos I. Tjonnfjord G. E. Kolstad A. Delabie J. Beiske K. Heim S. (2007). Molecular cytogenetic characterization of t(14;19)(q32;p13), a new recurrent translocation in B cell malignancies. Virchows Arch. 450, 559–565. 10.1007/s00428-007-0407-6 17406891

[B132] Michaud E. J. Yoder B. K. (2006). The primary cilium in cell signaling and cancer. Cancer Res. 66, 6463–6467. 10.1158/0008-5472.CAN-06-0462 16818613

[B133] Moustakas A. Souchelnytskyi S. Heldin C. H. (2001). Smad regulation in TGF-beta signal transduction. J. Cell Sci. 114, 4359–4369. 11792802 10.1242/jcs.114.24.4359

[B134] Mullor J. L. Dahmane N. Sun T. Ruiz i Altaba A. (2001). Wnt signals are targets and mediators of Gli function. Curr. Biol. 11, 769–773. 10.1016/S0960-9822(01)00229-9 11378387

[B135] Mumm J. S. Kopan R. (2000). Notch signaling: from the outside in. Dev. Biol. 228, 151–165. 10.1006/dbio.2000.9960 11112321

[B136] Napolitano M. Marfia G. A. Vacca A. Centonze D. Bellavia D. Di Marcotullio L. (1999). Modulation of gene expression following long-term synaptic depression in the striatum. Brain Res. Mol. Brain Res. 72, 89–96. 10.1016/S0169-328X(99)00213-2 10521602

[B137] Naylor T. L. Greshock J. Wang Y. Colligon T. Yu Q. C. Clemmer V. (2005). High resolution genomic analysis of sporadic breast cancer using array-based comparative genomic hybridization. Breast Cancer Res. 7, R1186–R1198. 10.1186/bcr1356 16457699 PMC1410746

[B138] Nessling M. Richter K. Schwaenen C. Roerig P. Wrobel G. Wessendorf S. (2005). Candidate genes in breast cancer revealed by microarray-based comparative genomic hybridization of archived tissue. Cancer Res. 65, 439–447. 15695385

[B139] Nilsson M. Unden A. B. Krause D. Malmqwist U. Raza K. Zaphiropoulos P. G. Toftgard R. (2000). Induction of basal cell carcinomas and trichoepitheliomas in mice overexpressing GLI-1. Proc. Natl Acad. Sci. U. S. A. 97, 3438–3443. 10.1073/pnas.97.7.3438 10725363 PMC16258

[B140] Nitzki F. Zibat A. Frommhold A. Schneider A. Schulz-Schaeffer W. Braun T., Hahn H. (2011). Uncommitted precursor cells might contribute to increased incidence of embryonal rhabdomyosarcoma in heterozygous Patched1-mutant mice. Oncogene 30, 4428–4436. 10.1038/onc.2011.157 21602886

[B141] Northcott P. A. Nakahara Y. Wu X. Feuk L. Ellison D. W. Croul S. (2009). Multiple recurrent genetic events converge on control of histone lysine methylation in medulloblastoma. Nat. Genet. 41, 465–472. 10.1038/ng.336 19270706 PMC4454371

[B142] Noubissi F. K. Goswami S. Sanek N. A. Kawakami K. Minamoto T. Moser A. (2009). Wnt signaling stimulates transcriptional outcome of the Hedgehog pathway by stabilizing GLI1 mRNA. Cancer Res. 69, 8572–8578. 10.1158/0008-5472.CAN-09-1500 19887615 PMC2783483

[B143] Nusslein-Volhard C. Wieschaus E. (1980). Mutations affecting segment number and polarity in *Drosophila*. Nature 287, 795–801. 10.1038/287795a0 6776413

[B144] Okuyama R. Tagami H. Aiba S. (2008). Notch signaling: its role in epidermal homeostasis and in the pathogenesis of skin diseases. J. Dermatol. Sci. 49, 187–194. 10.1016/j.jdermsci.2007.05.017 17624739

[B145] Oliver T. G. Grasfeder L. L. Carroll A. L. Kaiser C. Gillingham C. L. Lin S. M. (2003). Transcriptional profiling of the Sonic hedgehog response: a critical role for N-myc in proliferation of neuronal precursors. Proc. Natl Acad. Sci. U. S. A. 100, 7331–7336. 10.1073/pnas.0832317100 12777630 PMC165875

[B146] Ormestad M. Astorga J. Landgren H. Wang T. Johansson B. R. Miura N. Carlsson P. (2006). Foxf1 and Foxf2 control murine gut development by limiting mesenchymal Wnt signaling and promoting extracellular matrix production. Development 133, 833–843. 10.1242/dev.02252 16439479

[B147] Palermo R. Checquolo S. Bellavia D. Talora C. Screpanti I. (2014). The molecular basis of notch signaling regulation: a complex simplicity. Curr. Mol. Med. 14, 34–44. 10.2174/1566524013666131118105216 24236458

[B148] Palomero T. Lim W. K. Odom D. T. Sulis M. L. Real P. J. Margolin A. (2006). NOTCH1 directly regulates c-MYC and activates a feed-forward-loop transcriptional network promoting leukemic cell growth. Proc. Natl Acad. Sci. U. S. A. 103, 18261–18266. 10.1073/pnas.0606108103 17114293 PMC1838740

[B149] Pardali K. Kurisaki A. Moren A. Dijke P. Kardassis D. Moustakas A. (2000). Role of Smad proteins and transcription factor Sp1 in p21(Waf1/Cip1) regulation by transforming growth factor-beta. J. Biol. Chem. 275, 29244–29256. 10.1074/jbc.M909467199 10878024

[B150] Pearse R. V., LS Collier MP Scott Tabin C. J. (1999). Vertebrate homologs of *Drosophila* suppressor of fused interact with the gli family of transcriptional regulators. Dev. Biol. 212, 323–336. 10.1006/dbio.1999.9335 10433824 PMC4530617

[B151] Pelullo M. Quaranta R. Talora C. Checquolo S. Cialfi S. Felli M. P. (2014). Notch3/Jagged1 circuitry reinforces notch signaling and sustains T-ALL. Neoplasia 16, 1007–1017. 10.1016/j.neo.2014.10.004 25499214 PMC4309263

[B152] Petrova R. Joyner A. L. (2014). Roles for Hedgehog signaling in adult organ homeostasis and repair. Development 141, 3445–3457. 10.1242/dev.083691 25183867 PMC4197719

[B153] Pierrat M. J. Marsaud V. Mauviel A. Javelaud D. (2012). Expression of microphthalmia-associated transcription factor (MITF), which is critical for melanoma progression, is inhibited by both transcription factor GLI2 and transforming growth factor-beta. J. Biol. Chem. 287, 17996–18004. 10.1074/jbc.M112.358341 22496449 PMC3365743

[B154] Poschl J. Bartels M. Ohli J. Bianchi E. Kuteykin-Teplyakov K. Grammel D. (2014). Wnt/beta-catenin signaling inhibits the Shh pathway and impairs tumor growth in Shh-dependent medulloblastoma. Acta Neuropathol. 127, 605–607. 10.1007/s00401-014-1258-2 24531885

[B155] Price M. A. Kalderon D. (2002). Proteolysis of the Hedgehog signaling effector Cubitus interruptus requires phosphorylation by glycogen synthase kinase 3 and casein kinase 1. Cell 108, 823–835. 10.1016/S0092-8674(02)00664-5 11955435

[B156] Qualtrough D. Rees P. Speight B. Williams A. C. Paraskeva C. (2015). The Hedgehog inhibitor cyclopamine reduces beta-catenin–Tcf transcriptional activity, induces E-cadherin expression, and reduces invasion in colorectal cancer cells. Cancers 7, 1885–1899. 10.3390/cancers7030867 26393651 PMC4586800

[B157] Quaranta R. Pelullo M. Zema S. Nardozza F. Checquolo S. Lauer D. M. (2017). Maml1 acts cooperatively with Gli proteins to regulate sonic hedgehog signaling pathway. Cell Death Dis. 8, e2942. 10.1038/cddis.2017.326 28726779 PMC5550871

[B158] Radojcic V. Maillard I. (2014). A jagged road to lymphoma aggressiveness. Cancer cell 25, 261–263. 10.1016/j.ccr.2014.03.001 24651005 PMC4040245

[B159] Raffel C. Jenkins R. B. Frederick L. Hebrink D. Alderete B. Fults D. W. (1997). Sporadic medulloblastomas contain PTCH mutations. Cancer Res. 57 (5), 842–845. 9041183

[B160] Ramalho-Santos M. Melton D. A. McMahon A. P. (2000). Hedgehog signals regulate multiple aspects of gastrointestinal development. Development 127, 2763–2772. 10821773 10.1242/dev.127.12.2763

[B161] Reedijk M. Odorcic S. Zhang H. Chetty R. Tennert C. (2008). Activation of Notch signaling in human colon adenocarcinoma. Int. J. Oncol. 33, 1223–1229. 19020755 10.3892/ijo_00000112 PMC2739737

[B162] Regl G. Neill G. W. Eichberger T. Kasper M. Ikram M. S. Koller J. (2002). Human GLI2 and GLI1 are part of a positive feedback mechanism in Basal Cell Carcinoma. Oncogene 21, 5529–5539. 10.1038/sj.onc.1205748 12165851

[B163] Reifenberger J. Wolter M. Knobbe C. B. Kohler B. Schonicke A. Scharwachter C. (2005). Somatic mutations in the PTCH, SMOH, SUFUH and TP53 genes in sporadic basal cell carcinomas. Br. J. Dermatol. 152, 43–51. 10.1111/j.1365-2133.2005.06353.x 15656799

[B164] Reifenberger J. Wolter M. Weber R. G. Megahed M. Ruzicka T. Lichter P. (1998). Missense mutations in SMOH in sporadic basal cell carcinomas of the skin and primitive neuroectodermal tumors of the central nervous system. Cancer Res. 58, 1798–1803. 9581815

[B165] Rimkus T. K. Carpenter R. L. Qasem S. Chan M. Lo H. W. (2016). Targeting the sonic hedgehog signaling pathway: review of smoothened and GLI inhibitors. Cancers 8, 22–45. 10.3390/cancers8020022 PMC4773745 26891329

[B166] Roberts W. M. Douglass E. C. Peiper S. C. Houghton P. J. Look A. T. (1989). Amplification of the gli gene in childhood sarcomas. Cancer Res. 49, 5407–5413. 2766305

[B167] Rodilla V. Villanueva A. Obrador-Hevia A. Robert-Moreno A. Fernandez-Majada V. Grilli A. (2009). Jagged1 is the pathological link between Wnt and Notch pathways in colorectal cancer. Proc. Natl Acad. Sci. U. S. A. 106, 6315–6320. 10.1073/pnas.0813221106 19325125 PMC2669348

[B168] Rohatgi R. Milenkovic L. Scott M. P. (2007). Patched1 regulates hedgehog signaling at the primary cilium. Science 317, 372–376. 10.1126/science.1139740 17641202

[B169] Rosati E. Sabatini R. Rampino G. Tabilio A. Di Ianni M. Fettucciari K. (2009). Constitutively activated Notch signaling is involved in survival and apoptosis resistance of B-CLL cells. Blood 113, 856–865. 10.1182/blood-2008-02-139725 18796623

[B170] Ross D. A. Kadesch T. (2001). The notch intracellular domain can function as a coactivator for LEF-1. Mol. Cell. Biol. 21, 7537–7544. 10.1128/MCB.21.22.7537-7544.2001 11604490 PMC99925

[B171] Rowbotham N. J. Hager-Theodorides A. L. Furmanski A. L. Crompton T. (2007). A novel role for Hedgehog in T-cell receptor signaling: implications for development and immunity. Cell cycle 6, 2138–2142. 10.4161/cc.6.17.4644 17786048

[B172] Rusert J. M. Wu X. Eberhart C. G. Taylor M. D. Wechsler-Reya R. J. (2014). SnapShot: medulloblastoma. Cancer cell 26940-40, e1. 10.1016/j.ccell.2014.11.015 PMC4324613 25490452

[B173] Rustighi A. Zannini A. Tiberi L. Sommaggio R. Piazza S. Sorrentino G. (2014). Prolyl-isomerase Pin1 controls normal and cancer stem cells of the breast. EMBO Mol. Med. 6, 99–119. 10.1002/emmm.201302909 24357640 PMC3936488

[B174] Ryan K. E. Chiang C. (2012). Hedgehog secretion and signal transduction in vertebrates. J. Biol. Chem. 287, 17905–17913. 10.1074/jbc.R112.356006 22474285 PMC3365689

[B175] Sasaki H. Nishizaki Y. Hui C. Nakafuku M. Kondoh H. (1999). Regulation of Gli2 and Gli3 activities by an amino-terminal repression domain: implication of Gli2 and Gli3 as primary mediators of Shh signaling. Development 126, 3915–3924. 10433919 10.1242/dev.126.17.3915

[B176] Schreck K. C. Taylor P. Marchionni L. Gopalakrishnan V. Bar E. E. Gaiano N. Eberhart C. G. (2010). The Notch target Hes1 directly modulates Gli1 expression and Hedgehog signaling: a potential mechanism of therapeutic resistance. Clin. Cancer Res. 16, 6060–6070. 10.1158/1078-0432.CCR-10-1624 21169257 PMC3059501

[B177] Sengupta A. Banerjee D. Chandra S. Banerji S. K. Ghosh R. Roy R. (2007). Deregulation and cross talk among Sonic hedgehog, Wnt, Hox and Notch signaling in chronic myeloid leukemia progression. Leukemia 21, 949–955. 10.1038/sj.leu.2404657 17361218

[B178] Seoane J. Pouponnot C. Staller P. Schader M. Eilers M. Massague J. (2001). TGFbeta influences Myc, Miz-1 and Smad to control the CDK inhibitor p15INK4b. Nat. Cell Biol. 3, 400–408. 10.1038/35070086 11283614

[B179] Sheng T. Li C. Zhang X. Chi S. He N. Chen K. (2004). Activation of the hedgehog pathway in advanced prostate cancer. Mol. Cancer 3, 29. 10.1186/1476-4598-3-29 15482598 PMC524523

[B180] Shin H. M. Minter L. M. Cho O. H. Gottipati S. Fauq A. H. Golde T. E. (2006). Notch1 augments NF-kappaB activity by facilitating its nuclear retention. EMBO J. 25, 129–138. 10.1038/sj.emboj.7600902 16319921 PMC1356346

[B181] Siegel P. M. Massague J. (2003). Cytostatic and apoptotic actions of TGF-beta in homeostasis and cancer. Nat. Rev. Cancer 3, 807–821. 10.1038/nrc1208 14557817

[B182] Sikandar S. S. Pate K. T. Anderson S. Dizon D. Edwards R. A. Waterman M. L. (2010). NOTCH signaling is required for formation and self-renewal of tumor-initiating cells and for repression of secretory cell differentiation in colon cancer. Cancer Res. 70, 1469–1478. 10.1158/0008-5472.CAN-09-2557 20145124 PMC4010106

[B183] Singh R. R. Kim J. E. Davuluri Y. Drakos E. Cho-Vega J. H. Amin H. M. (2010). Hedgehog signaling pathway is activated in diffuse large B-cell lymphoma and contributes to tumor cell survival and proliferation. Leukemia 24, 1025–1036. 10.1038/leu.2010.35 20200556

[B184] Sinha S. Singh R. K. Alam N. Roy A. Roychoudhury S. Panda C. K. (2008). Frequent alterations of hMLH1 and RBSP3/HYA22 at chromosomal 3p22.3 region in early and late-onset breast carcinoma: clinical and prognostic significance. Cancer Sci. 99, 1984–1991. 10.1111/j.1349-7006.2008.00952.x 19016758 PMC11158254

[B185] Sisson B. E. Ziegenhorn S. L. Holmgren R. A. (2006). Regulation of Ci and Su(fu) nuclear import in *Drosophila*. Dev. Biol. 294, 258–270. 10.1016/j.ydbio.2006.02.050 16595130

[B186] Smith M. J. Beetz C. Williams S. G. Bhaskar S. S. O’Sullivan J. Anderson B. (2014). Germline mutations in SUFU cause Gorlin syndrome–associated childhood medulloblastoma and redefine the risk associated with PTCH1 mutations. J. Clin. Oncol. 32, 4155–4161. 10.1200/JCO.2014.58.2569 25403219

[B187] Staal F. J. Famili F. Garcia Perez L. Pike-Overzet K. (2016). Aberrant Wnt signaling in leukemia. Cancers 8, 78–93. 10.3390/cancers8090078 PMC5040980 27571104

[B188] Stasiulewicz M. Gray S. D. Mastromina I. Silva J. C. Bjorklund M. Seymour P. A. (2015). A conserved role for Notch signaling in priming the cellular response to Shh through ciliary localisation of the key Shh transducer Smo. Development 142, 2291–2303. 10.1242/dev.125237 25995356 PMC4510595

[B189] Stecca B. Ruiz i Altaba A. (2009). A GLI1–p53 inhibitory loop controls neural stem cell and tumour cell numbers. EMBO J. 28, 663–676. 10.1038/emboj.2009.16 19214186 PMC2647769

[B190] Steg A. D. Katre A. A. Goodman B. Han H. D. Nick A. M. Stone R. L. (2011). Targeting the notch ligand JAGGED1 in both tumor cells and stroma in ovarian cancer. Clin. Cancer Res. 17, 5674–5685. 10.1158/1078-0432.CCR-11-0432 21753153 PMC3166981

[B191] Stone D. M. Murone M. Luoh S. Ye W. Armanini M. P. Gurney A. (1999). Characterization of the human suppressor of fused, a negative regulator of the zinc-finger transcription factor Gli. J. Cell Sci. 112 (Pt 23), 4437–4448. 10564661 10.1242/jcs.112.23.4437

[B192] Swartling F. J. Grimmer M. R. Hackett C. S. Northcott P. A. Fan Q. W. Goldenberg D. D. (2010). Pleiotropic role for MYCN in medulloblastoma. Genes Dev. 24, 1059–1072. 10.1101/gad.1907510 20478998 PMC2867210

[B193] Sweeney R. T. McClary A. C. Myers B. R. Biscocho J. Neahring L. Kwei K. A. (2014). Identification of recurrent SMO and BRAF mutations in ameloblastomas. Nat. Genet. 46, 722–725. 10.1038/ng.2986 24859340 PMC4418232

[B194] Syed V. (2016). TGF-beta signaling in cancer. J. Cell. Biochem. 117, 1279–1287. 10.1002/jcb.25496 26774024

[B195] Taeubner J. Brozou T. Qin N. Bartl J. Ginzel S. Schaper J. (2018). Congenital embryonal rhabdomyosarcoma caused by heterozygous concomitant PTCH1 and PTCH2 germline mutations. Eur. J. Human Genet. 26, 137–142. 10.1038/s41431-017-0048-4 29230040 PMC5839031

[B196] Taipale J. Cooper M. K. Maiti T. Beachy P. A. (2002). Patched acts catalytically to suppress the activity of Smoothened. Nature 418, 892–897. 10.1038/nature00989 12192414

[B197] Takebe N. Harris P. J. Warren R. Q. Ivy S. P. (2011). Targeting cancer stem cells by inhibiting Wnt, Notch, and Hedgehog pathways. Nat. Rev. Clin. Oncol. 8, 97–106. 10.1038/nrclinonc.2010.196 21151206

[B198] Talora C. Campese A. F. Bellavia D. Felli M. P. Vacca A. Gulino A. (2008). Notch signaling and diseases: an evolutionary journey from a simple beginning to complex outcomes. Biochim. Biophys. Acta 1782, 489–497. 10.1016/j.bbadis.2008.06.008 18625307

[B199] Talora C. Cialfi S. Oliviero C. Palermo R. Pascucci M. Frati L. (2006). Cross talk among Notch3, pre-TCR, and Tal1 in T-cell development and leukemogenesis. Blood 107, 3313–3320. 10.1182/blood-2005-07-2823 16368887

[B200] Taylor M. D. Liu L. Raffel C. Hui C. C. Mainprize T. G. Zhang X. (2002). Mutations in SUFU predispose to medulloblastoma. Nat. Genet. 31, 306–310. 10.1038/ng916 12068298

[B201] Taylor M. D. Zhang X. Liu L. Hui C. C. Mainprize T. G. Scherer S. W. (2004). Failure of a medulloblastoma-derived mutant of SUFU to suppress WNT signaling. Oncogene 23, 4577–4583. 10.1038/sj.onc.1207605 15077159

[B202] Teglund S. Toftgard R. (2010). Hedgehog beyond medulloblastoma and basal cell carcinoma. Biochim. Biophys. Acta 1805, 181–208. 10.1016/j.bbcan.2010.01.003 20085802

[B203] Tetsu O. McCormick F. (1999). Beta-catenin regulates expression of cyclin D1 in colon carcinoma cells. Nature 398, 422–426. 10.1038/18884 10201372

[B204] Thayer S. P. di Magliano M. P. Heiser P. W. Nielsen C. M. Roberts D. J. Lauwers G. Y. (2003). Hedgehog is an early and late mediator of pancreatic cancer tumorigenesis. Nature 425, 851–856. 10.1038/nature02009 14520413 PMC3688051

[B205] Thomas W. D. Chen J. Gao Y. R. Cheung B. Koach J. Sekyere E. (2009). Patched1 deletion increases N-Myc protein stability as a mechanism of medulloblastoma initiation and progression. Oncogene 28, 1605–1615. 10.1038/onc.2009.3 19234491

[B206] Thompson M. C. Fuller C. Hogg T. L. Dalton J. Finkelstein D. Lau C. C. (2006). Genomics identifies medulloblastoma subgroups that are enriched for specific genetic alterations. J. Clin. Oncol. 24, 1924–1931. 10.1200/JCO.2005.04.4974 16567768

[B207] Tottone L. Zhdanovskaya N. Carmona Pestana A. Zampieri M. Simeoni F. Lazzari S. (2019). Histone modifications drive aberrant Notch3 expression/activity and growth in T-ALL. Front. Oncol. 9, 198. 10.3389/fonc.2019.00198 31001470 PMC6456714

[B208] Ulloa F. Itasaki N. Briscoe J. (2007). Inhibitory Gli3 activity negatively regulates Wnt/beta-catenin signaling. Curr. Biol. 17, 545–550. 10.1016/j.cub.2007.01.062 17331723

[B209] Vacca A. Felli M. P. Palermo R. Di Mario G. Calce A. Di Giovine M. (2006). Notch3 and pre-TCR interaction unveils distinct NF-kappaB pathways in T-cell development and leukemia. EMBO J. 25, 1000–1008. 10.1038/sj.emboj.7600996 16498412 PMC1409728

[B210] van den Brink G. R. Bleuming S. A. Hardwick J. C. Schepman B. L. Offerhaus G. J. Keller J. J. (2004). Indian Hedgehog is an antagonist of Wnt signaling in colonic epithelial cell differentiation. Nat. Genet. 36, 277–282. 10.1038/ng1304 14770182

[B211] Vargas Romero P. Cialfi S. Palermo R. De Blasio C. Checquolo S. Bellavia D. (2015). The deregulated expression of miR-125b in acute myeloid leukemia is dependent on the transcription factor C/EBPalpha. Leukemia 29, 2442–2445. 10.1038/leu.2015.117 25982911 PMC4675867

[B212] Varjosalo M. Taipale J. (2008). Hedgehog: functions and mechanisms. Genes Dev. 22, 2454–2472. 10.1101/gad.1693608 18794343

[B213] Varnat F. Duquet A. Malerba M. Zbinden M. Mas C. Gervaz P. Ruiz i Altaba A. (2009). Human colon cancer epithelial cells harbour active HEDGEHOG-GLI signalling that is essential for tumour growth, recurrence, metastasis and stem cell survival and expansion. EMBO Mol. Med. 1, 338–351. 10.1002/emmm.200900039 20049737 PMC3378144

[B214] Varnat F. Siegl-Cachedenier I. Malerba M. Gervaz P. Ruiz i Altaba A. (2010). Loss of WNT–TCF addiction and enhancement of HH-GLI1 signalling define the metastatic transition of human colon carcinomas. EMBO Mol. Med. 2, 440–457. 10.1002/emmm.201000098 20941789 PMC3394505

[B215] Varnum-Finney B. Xu L. Brashem-Stein C. Nourigat C. Flowers D. Bakkour S. (2000). Pluripotent, cytokine-dependent, hematopoietic stem cells are immortalized by constitutive Notch1 signaling. Nat. Med. 6, 1278–1281. 10.1038/81390 11062542

[B216] Verrecchia F. Mauviel A. (2007). Transforming growth factor-beta and fibrosis. World J. Gastroenterol. 13, 3056–3062. 10.3748/wjg.v13.i22.3056 17589920 PMC4172611

[B217] Vied C. Kalderon D. (2009). Hedgehog-stimulated stem cells depend on non-canonical activity of the Notch co-activator Mastermind. Development 136, 2177–2186. 10.1242/dev.035329 19474148 PMC2729338

[B218] Vokes S. A. Ji H. McCuine S. Tenzen T. Giles S. Zhong S. (2007). Genomic characterization of Gli-activator targets in sonic hedgehog–mediated neural patterning. Development 134, 1977–1989. 10.1242/dev.001966 17442700

[B219] Wahl S. M. (1994). Transforming growth factor beta: the good, the bad, and the ugly. J. Exp. Med. 180, 1587–1590. 10.1084/jem.180.5.1587 7964446 PMC2191721

[B220] Wall D. S. Mears A. J. McNeill B. Mazerolle C. Thurig S. Wang Y. (2009). Progenitor cell proliferation in the retina is dependent on Notch-independent Sonic hedgehog/Hes1 activity. J. Cell Biol. 184, 101–112. 10.1083/jcb.200805155 19124651 PMC2615087

[B221] Wang X. D. Inzunza H. Chang H. Qi Z. Hu B. Malone D. Cogswell J. (2013). Mutations in the hedgehog pathway genes SMO and PTCH1 in human gastric tumors. PloS One 8, e54415. 10.1371/journal.pone.0054415 23349881 PMC3548780

[B222] Weng A. P. Millholland J. M. Yashiro-Ohtani Y. Arcangeli M. L. Lau A. Wai C. (2006). c-Myc is an important direct target of Notch1 in T-cell acute lymphoblastic leukemia/lymphoma. Genes Dev. 20, 2096–2109. 10.1101/gad.1450406 16847353 PMC1536060

[B223] Wiemels J. Wrensch M. Claus E. B. (2010). Epidemiology and etiology of meningioma. J. Neuro-oncol. 99, 307–314. 10.1007/s11060-010-0386-3 PMC2945461 20821343

[B224] Wils L. J. Bijlsma M. F. (2018). Epigenetic regulation of the Hedgehog and Wnt pathways in cancer. Crit. Rev. Oncol. Hematol. 121, 23–44. 10.1016/j.critrevonc.2017.11.013 29279097

[B225] Wolfraim L. A. Fernandez T. M. Mamura M. Fuller W. L. Kumar R. Cole D. E. (2004). Loss of Smad3 in acute T-cell lymphoblastic leukemia. N. Engl. J. Med. 351, 552–559. 10.1056/NEJMoa031197 15295048

[B226] Wong S. Y. Reiter J. F. (2008). The primary cilium at the crossroads of mammalian hedgehog signaling. Curr. Top. Dev. Biol. 85, 225–260. 10.1016/S0070-2153(08)00809-0 19147008 PMC2653622

[B227] Wood L. D. Parsons D. W. Jones S. Lin J. Sjoblom T. Leary R. J. (2007). The genomic landscapes of human breast and colorectal cancers. Science 318, 1108–1113. 10.1126/science.1145720 17932254

[B228] Wu L. Aster J. C. Blacklow S. C. Lake R. Artavanis-Tsakonas S. Griffin J. D. (2000). MAML1, a human homologue of *Drosophila* mastermind, is a transcriptional co-activator for NOTCH receptors. Nat. Genet. 26, 484–489. 10.1038/82644 11101851

[B229] Xie G. Karaca G. Swiderska-Syn M. Michelotti G. A. Kruger L. Chen Y. (2013). Cross-talk between Notch and Hedgehog regulates hepatic stellate cell fate in mice. Hepatology 58, 1801–1813. 10.1002/hep.26511 23703657 PMC3758784

[B230] Xie J. Murone M. Luoh S. M. Ryan A. Gu Q. Zhang C. (1998). Activating Smoothened mutations in sporadic basal-cell carcinoma. Nature 391, 90–92. 10.1038/34201 9422511

[B231] Yang X. Letterio J. J. Lechleider R. J. Chen L. Hayman R. Gu H. (1999). Targeted disruption of SMAD3 results in impaired mucosal immunity and diminished T cell responsiveness to TGF-beta. EMBO J. 18, 1280–1291. 10.1093/emboj/18.5.1280 10064594 PMC1171218

[B232] Yavropoulou M. P. Maladaki A. Yovos J. G. (2015). The role of Notch and Hedgehog signaling pathways in pituitary development and pathogenesis of pituitary adenomas. Hormones 14, 5–18. 10.1007/BF03401377 25885100

[B233] Yoshikawa K. Shimada M. Miyamoto H. Higashijima J. Miyatani T. Nishioka M. (2009). Sonic hedgehog relates to colorectal carcinogenesis. J. Gastroenterol. 44, 1113–1117. 10.1007/s00535-009-0110-2 19662327

[B234] Yoshimoto A. N. Bernardazzi C. Carneiro A. J. Elia C. C. Martinusso C. A. Ventura G. M. (2012). Hedgehog pathway signaling regulates human colon carcinoma HT-29 epithelial cell line apoptosis and cytokine secretion. PloS One 7, e45332. 10.1371/journal.pone.0045332 23028941 PMC3446889

[B235] Zavadil J. Bottinger E. P. (2005). TGF-beta and epithelial-to-mesenchymal transitions. Oncogene 24, 5764–5774. 10.1038/sj.onc.1208927 16123809

[B236] Zavadil J. Cermak L. Soto-Nieves N. Bottinger E. P. (2004). Integration of TGF-beta/Smad and Jagged1/Notch signalling in epithelial-to-mesenchymal transition. EMBO J. 23, 1155–1165. 10.1038/sj.emboj.7600069 14976548 PMC380966

[B237] Zi Z. Chapnick D. A. Liu X. (2012). Dynamics of TGF-beta/Smad signaling. FEBS Lett. 586, 1921–1928. 10.1016/j.febslet.2012.03.063 22710166 PMC4127320

[B238] Zinke J. Schneider F. T. Harter P. N. Thom S. Ziegler N. Toftgard R. (2015). Beta-catenin–Gli1 interaction regulates proliferation and tumor growth in medulloblastoma. Mol. Cancer 14, 17. 10.1186/s12943-015-0294-4 25645196 PMC4320815

[B239] Zurawel R. H. Allen C. Chiappa S. Cato W. Biegel J. Cogen P. (2000). Analysis of PTCH/SMO/SHH pathway genes in medulloblastoma. Genes Chromosomes Cancer 27, 44–51. 10.1002/(SICI)1098-2264(200001)27:1<44::AID-GCC6>3.0.CO;2-V 10564585

